# Supplementary material for: Temporally controlled multistep division of DNA droplets for dynamic artificial cells
Source: Nat Commun. 2024 Aug 27;15:7397. doi: 10.1038/s41467-024-51299-5 (PMC11350047; doi:10.1038/s41467-024-51299-5)
Supplement: Supplementary file 1 — Supplementary Information [file 41467_2024_51299_MOESM1_ESM.pdf]

Supplementary information

Temporally controlled multistep division of DNA droplets for dynamic artificial cells

Tomoya Maruyama[1], Jing Gong[1], Masahiro Takinoue[1,2,3,*]

[1] Department of Life Science and Technology, Tokyo Institute of Technology, 4259 Nagatsuta-cho, Midori-ku, Yokohama, Kanagawa 226-8501, Japan
[2] Department of Computer Science, Tokyo Institute of Technology, 4259 Nagatsuta-cho, Midori-ku, Yokohama, Kanagawa 226-8501, Japan
[3] Research Center for Autonomous Systems Materialogy (ASMat), Institute of Innovative Research, Tokyo Institute of Technology, 4259 Nagatsuta-cho, Midori-ku, Yokohama, Kanagawa 226-8501, Japan

*E-mail: takinoue@c.titech.ac.jp

Table of Contents

- Supplementary Notes 1-3
- Supplementary Figures 1-7
- Supplementary Tables 1-11
- Supplementary Movies 1-10

Supplementary Note

Supplementary Note 1: Reaction-diffusion simulation of Linker cleavage reactions of L_{AB} and $L^{\dagger}_{AB}$

A. Schematic of full numerical simulation model

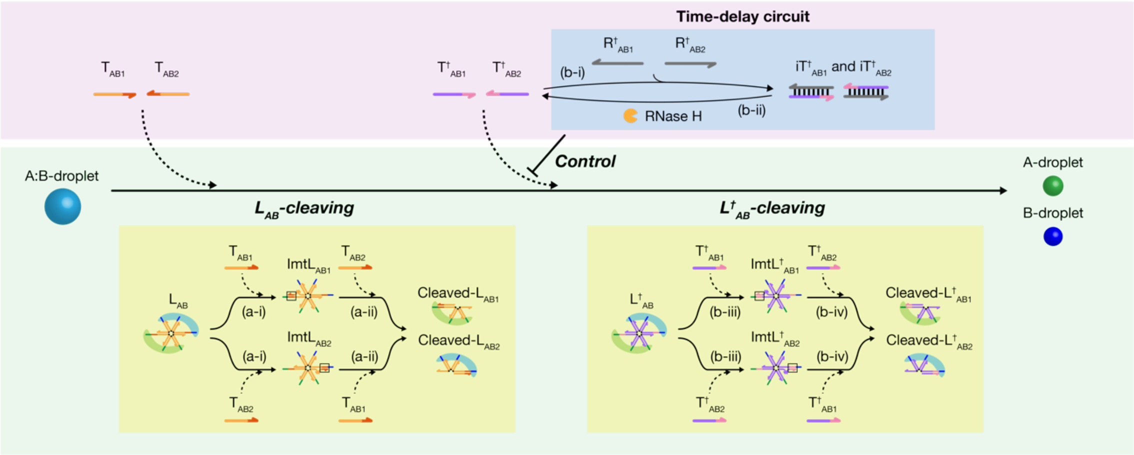

B. Rate equation

The cleavage of L_{AB} was modeled using reactions (a-i) and (a-ii). (a-i) The active division triggers (T_{AB1} and T_{AB2}) first hybridize with L_{AB} and intermediate complexes ($ItmL_{AB1}$ and $ItmL_{AB2}$) are produced. (a-ii) After hybridization, $ItmL_{AB1}$ and $ItmL_{AB2}$ were hybridized with T_{AB2} and T_{AB1}, respectively, and then cleaved into linker DNA particles (Cleaved-L_{AB1} and Cleaved-L_{AB2}) by the strand displacement reaction. Reactions (a-i) are as follows:

$$\mathrm{L_{AB}} + \mathrm{T_{AB1}} \overset{k_{\mathrm{h_{AB}}}}{\longrightarrow} \mathrm{ItmL_{AB1}}\,, \tag{S.1}$$

$$\mathrm{L_{AB}} + \mathrm{T_{AB2}} \overset{k_{\mathrm{h_{AB}}}}{\longrightarrow} \mathrm{ItmL_{AB2}}\,, \tag{S.2}$$

where $k_{\mathrm{h_{AB}}}$ is the rate constant of hybridization between the trigger and L_{AB}. Reactions (a-ii) are as follows:

$$\mathrm{ItmL_{AB1}} + \mathrm{T_{AB2}} \overset{k_{\mathrm{SD_{AB}}}}{\longrightarrow} \mathrm{ClvL_{AB1}} + \mathrm{ClvL_{AB2}}\,, \tag{S.3}$$

$$\mathrm{ItmL_{AB2}} + \mathrm{T_{AB1}} \overset{k_{\mathrm{SD_{AB}}}}{\longrightarrow} \mathrm{ClvL_{AB1}} + \mathrm{ClvL_{AB2}}\,, \tag{S.4}$$

where $k_{\mathrm{SD_{AB}}}$ represents the rate constant of the strand-displacement reaction for L_{AB} and $\mathrm{ClvL_{AB1}}$ and $\mathrm{ClvL_{AB2}}$ are Cleaved-L_{AB1} and Cleaved-L_{AB2}, respectively.

A time-delay circuit for $L^{\dagger}_{AB}$ is modeled by reactions, (b-i) and (b-ii). (b-i) The excess single-stranded RNAs (ssRNAs), named inhibitor RNAs ($R^{\dagger}_{AB1}$ and $R^{\dagger}_{AB2}$), hybridize with active division triggers ($T^{\dagger}_{AB1}$ and $T^{\dagger}_{AB2}$) and generate inhibited triggers ($iT^{\dagger}_{AB1}$ and $iT^{\dagger}_{AB2}$), respectively. (b-ii) Active division triggers ($T^{\dagger}_{AB1}$ and $T^{\dagger}_{AB2}$) are generated from inhibited triggers ($iT^{\dagger}_{AB1}$ and $iT^{\dagger}_{AB2}$) by an enzymatic RNA degradation with RNase H, respectively. These reactions cause the time delay of the expression of active division triggers, leading to the delay of $L^{\dagger}_{AB}$ cleavage. Reactions (b-i) are as follows:

$$\mathrm{T^{\dagger}}_{\mathrm{AB1}} + \mathrm{R^{\dagger}}_{\mathrm{AB1}} \overset{k_{\mathrm{h_{RNA}}}}{\longrightarrow} \mathrm{iT^{\dagger}}_{\mathrm{AB1}}\,, \tag{S.5}$$

$$\mathrm{T^{\dagger}}_{\mathrm{AB2}} + \mathrm{R^{\dagger}}_{\mathrm{AB2}} \overset{k_{\mathrm{h_{RNA}}}}{\longrightarrow} \mathrm{iT^{\dagger}}_{\mathrm{AB2}}\,, \tag{S.6}$$

where $k_{\mathrm{h_{RNA}}}$ represents the rate constant of the hybridization reaction between the active division triggers and the inhibitor RNAs. Reactions (b-ii) are represented using the Michaelis-Menten model as follows:

$$\mathrm{iT^{\dagger}}_{\mathrm{AB1}} + \mathrm{E_{RH}} \underset{k_2}{\overset{k_1}{\rightleftarrows}} \mathrm{iT^{\dagger}}_{\mathrm{AB1}}\mathrm{E_{RH}} \overset{k_{\mathrm{cat}}}{\longrightarrow} \mathrm{T^{\dagger}}_{\mathrm{AB1}} + \mathrm{E_{RH}}\,, \tag{S.7}$$

$$\mathrm{iT^{\dagger}}_{\mathrm{AB2}} + \mathrm{E_{RH}} \underset{k_2}{\overset{k_1}{\rightleftarrows}} \mathrm{iT^{\dagger}}_{\mathrm{AB2}}\mathrm{E_{RH}} \overset{k_{\mathrm{cat}}}{\longrightarrow} \mathrm{T^{\dagger}}_{\mathrm{AB2}} + \mathrm{E_{RH}}\,, \tag{S.8}$$

where $\mathrm{iT^{\dagger}}_{\mathrm{AB1}}\mathrm{E_{RH}}$ and $\mathrm{iT^{\dagger}}_{\mathrm{AB2}}\mathrm{E_{RH}}$ are the enzyme-substrate complexes; $\mathrm{E_{RH}}$ is RNase H; k_1, k_2, k_{cat} represent the rate constants of RNase H reaction.

The cleavage reaction of $L^{\dagger}_{AB}$ is modeled by reactions (b-iii) and (b-iv) in the same manner as the cleavage reactions of L_{AB}. Reactions (b-iii) and (b-iv) can be described as follows:

$$\mathrm{L^{\dagger}}_{\mathrm{AB}} + \mathrm{T^{\dagger}}_{\mathrm{AB1}} \overset{k_{\mathrm{h_{AB}}}}{\longrightarrow} \mathrm{ItmL^{\dagger}}_{\mathrm{AB1}}\,, \tag{S.9}$$

$$\mathrm{L^{\dagger}}_{\mathrm{AB}} + \mathrm{T^{\dagger}}_{\mathrm{AB2}} \overset{k_{\mathrm{h_{AB}}}}{\longrightarrow} \mathrm{ItmL^{\dagger}}_{\mathrm{AB2}}\,, \tag{S.10}$$

$$\mathrm{ItmL^{\dagger}}_{\mathrm{AB1}} + \mathrm{T^{\dagger}}_{\mathrm{AB2}} \overset{k_{\mathrm{SD_{AB}}}}{\longrightarrow} \mathrm{ClvL^{\dagger}}_{\mathrm{AB1}} + \mathrm{ClvL^{\dagger}}_{\mathrm{AB2}}\,, \tag{S.11}$$

$$\mathrm{ItmL^{\dagger}}_{\mathrm{AB2}} + \mathrm{T^{\dagger}}_{\mathrm{AB1}} \overset{k_{\mathrm{SD_{AB}}}}{\longrightarrow} \mathrm{ClvL^{\dagger}}_{\mathrm{AB1}} + \mathrm{ClvL^{\dagger}}_{\mathrm{AB2}}\,, \tag{S.12}$$

Based on these chemical reaction equations, the rate equation for each molecule is as follows:

$$\frac{du_{\mathrm{L_{AB}}}}{dt} = -k_{\mathrm{h_{AB}}} u_{\mathrm{L_{AB}}} u_{\mathrm{T_{AB1}}} - k_{\mathrm{h_{AB}}} u_{\mathrm{L_{AB}}} u_{\mathrm{T_{AB2}}} \, , \tag{S.13}$$

$$\frac{du_{\mathrm{T_{AB1}}}}{dt} = -k_{\mathrm{h_{AB}}} u_{\mathrm{L_{AB}}} u_{\mathrm{T_{AB1}}} - k_{\mathrm{SD_{AB}}} u_{\mathrm{ImtL_{AB2}}} u_{\mathrm{T_{AB1}}} \, , \tag{S.14}$$

$$\frac{du_{\mathrm{T_{AB2}}}}{dt} = -k_{\mathrm{h_{AB}}} u_{\mathrm{L_{AB}}} u_{\mathrm{T_{AB2}}} - k_{\mathrm{SD_{AB}}} u_{\mathrm{ImtL_{AB1}}} u_{\mathrm{T_{AB2}}} \, , \tag{S.15}$$

$$\frac{du_{\mathrm{ImtL_{AB1}}}}{dt} = k_{\mathrm{h_{AB}}} u_{\mathrm{L_{AB}}} u_{\mathrm{T_{AB1}}} - k_{\mathrm{SD_{AB}}} u_{\mathrm{ImtL_{AB1}}} u_{\mathrm{T_{AB2}}} \, , \tag{S.16}$$

$$\frac{du_{\mathrm{ImtL_{AB2}}}}{dt} = k_{\mathrm{h_{AB}}} u_{\mathrm{L_{AB}}} u_{\mathrm{T_{AB2}}} - k_{\mathrm{SD_{AB}}} u_{\mathrm{ImtL_{AB2}}} u_{\mathrm{T_{AB1}}} \, , \tag{S.17}$$

$$\frac{du_{\mathrm{ClvL_{AB1}}}}{dt} = k_{\mathrm{SD_{AB}}} u_{\mathrm{ImtL_{AB1}}} u_{\mathrm{T_{AB2}}} + k_{\mathrm{SD_{AB}}} u_{\mathrm{ImtL_{AB2}}} u_{\mathrm{T_{AB1}}} \, , \tag{S.18}$$

$$\frac{du_{\mathrm{ClvL_{AB2}}}}{dt} = k_{\mathrm{SD_{AB}}} u_{\mathrm{ImtL_{AB1}}} u_{\mathrm{T_{AB2}}} + k_{\mathrm{SD_{AB}}} u_{\mathrm{ImtL_{AB2}}} u_{\mathrm{T_{AB1}}} \, , \tag{S.19}$$

$$\frac{du_{\mathrm{iT^{\dagger}{}_{AB1}}}}{dt} = -\frac{k_{\mathrm{cat}} c_{\mathrm{E_{RH}}} u_{\mathrm{iT^{\dagger}{}_{AB1}}}}{K_{\mathrm{m}} + u_{\mathrm{iT^{\dagger}{}_{AB1}}}} + k_{\mathrm{h_{RNA}}} u_{\mathrm{T^{\dagger}{}_{AB1}}} u_{\mathrm{R^{\dagger}{}_{AB1}}} \, , \tag{S.20}$$

$$\frac{du_{\mathrm{iT^{\dagger}{}_{AB2}}}}{dt} = -\frac{k_{\mathrm{cat}} c_{\mathrm{E_{RH}}} u_{\mathrm{iT^{\dagger}{}_{AB2}}}}{K_{\mathrm{m}} + u_{\mathrm{iT^{\dagger}{}_{AB2}}}} + k_{\mathrm{h_{RNA}}} u_{\mathrm{T^{\dagger}{}_{AB2}}} u_{\mathrm{R^{\dagger}{}_{AB2}}} \, , \tag{S.21}$$

$$\frac{du_{\mathrm{R^{\dagger}{}_{AB1}}}}{dt} = -k_{\mathrm{h_{RNA}}} u_{\mathrm{T^{\dagger}{}_{AB1}}} u_{\mathrm{R^{\dagger}{}_{AB1}}} \, , \tag{S.22}$$

$$\frac{du_{\mathrm{R^{\dagger}{}_{AB2}}}}{dt} = -k_{\mathrm{h_{RNA}}} u_{\mathrm{T^{\dagger}{}_{AB2}}} u_{\mathrm{R^{\dagger}{}_{AB2}}} \, , \tag{S.23}$$

$$\frac{du_{\mathrm{L^{\dagger}{}_{AB}}}}{dt} = -k_{\mathrm{h_{AB}}} u_{\mathrm{L^{\dagger}{}_{AB}}} u_{\mathrm{T^{\dagger}{}_{AB1}}} - k_{\mathrm{h_{AB}}} u_{\mathrm{L^{\dagger}{}_{AB}}} u_{\mathrm{T^{\dagger}{}_{AB2}}} \, , \tag{S.24}$$

$$\begin{aligned} \frac{du_{\mathrm{T^{\dagger}{}_{AB1}}}}{dt} &= \frac{k_{\mathrm{cat}} c_{\mathrm{E_{RH}}} u_{\mathrm{iT^{\dagger}{}_{AB1}}}}{K_{\mathrm{m}} + u_{\mathrm{iT^{\dagger}{}_{AB1}}}} - k_{\mathrm{h_{RNA}}} u_{\mathrm{T^{\dagger}{}_{AB1}}} u_{\mathrm{R^{\dagger}{}_{AB1}}} \\ &\quad -k_{\mathrm{h_{AB}}} u_{\mathrm{L^{\dagger}{}_{AB}}} u_{\mathrm{T^{\dagger}{}_{AB1}}} - k_{\mathrm{SD_{AB}}} u_{\mathrm{ImtL^{\dagger}{}_{AB2}}} u_{\mathrm{T^{\dagger}{}_{AB1}}} \, , \end{aligned} \tag{S.25}$$

$$\begin{aligned} \frac{du_{\mathrm{T^{\dagger}{}_{AB2}}}}{dt} &= \frac{k_{\mathrm{cat}} c_{\mathrm{E_{RH}}} u_{\mathrm{iT^{\dagger}{}_{AB2}}}}{K_{\mathrm{m}} + u_{\mathrm{iT^{\dagger}{}_{AB2}}}} - k_{\mathrm{h_{RNA}}} u_{\mathrm{T^{\dagger}{}_{AB2}}} u_{\mathrm{R^{\dagger}{}_{AB2}}} \\ &\quad -k_{\mathrm{h_{AB}}} u_{\mathrm{L^{\dagger}{}_{AB}}} u_{\mathrm{T^{\dagger}{}_{AB2}}} - k_{\mathrm{SD_{AB}}} u_{\mathrm{ImtL^{\dagger}{}_{AB1}}} u_{\mathrm{T^{\dagger}{}_{AB2}}} \, , \end{aligned} \tag{S.26}$$

$$\frac{du_{\mathrm{ImtL^{\dagger}{}_{AB1}}}}{dt} = k_{\mathrm{h_{AB}}} u_{\mathrm{L^{\dagger}{}_{AB}}} u_{\mathrm{T^{\dagger}{}_{AB1}}} - k_{\mathrm{SD_{AB}}} u_{\mathrm{ImtL^{\dagger}{}_{AB1}}} u_{\mathrm{T^{\dagger}{}_{AB2}}} \, , \tag{S.27}$$

$$\frac{du_{\mathrm{ImtL^{\dagger}{}_{AB2}}}}{dt} = k_{\mathrm{h_{AB}}} u_{\mathrm{L^{\dagger}{}_{AB}}} u_{\mathrm{T^{\dagger}{}_{AB2}}} - k_{\mathrm{SD_{AB}}} u_{\mathrm{ImtL^{\dagger}{}_{AB2}}} u_{\mathrm{T^{\dagger}{}_{AB1}}} \, , \tag{S.28}$$

$$\frac{du_{\mathrm{ClvL^{\dagger}{}_{AB1}}}}{dt} = k_{\mathrm{SD_{AB}}} u_{\mathrm{ImtL^{\dagger}{}_{AB1}}} u_{\mathrm{T^{\dagger}{}_{AB2}}} + k_{\mathrm{SD_{AB}}} u_{\mathrm{ImtL^{\dagger}{}_{AB2}}} u_{\mathrm{T^{\dagger}{}_{AB1}}} \, , \tag{S.29}$$

$$\frac{du_{\mathrm{ClvL^{\dagger}}_{\mathrm{AB2}}}}{dt} = k_{\mathrm{SD}_{\mathrm{AB}}} u_{\mathrm{ImtL^{\dagger}}_{\mathrm{AB1}}} u_{\mathrm{T^{\dagger}}_{\mathrm{AB2}}} + k_{\mathrm{SD}_{\mathrm{AB}}} u_{\mathrm{ImtL^{\dagger}}_{\mathrm{AB2}}} u_{\mathrm{T^{\dagger}}_{\mathrm{AB1}}}\,, \tag{S.30}$$

where u_{X} is the concentration of molecule 'X', and $c_{\mathrm{E_{RH}}}$ is the initial concentration of RNase H. K_{m} is the Michaelis-Menten parameter and is described as follows:

$$K_{\mathrm{m}} = \frac{k_2 + k_{\mathrm{cat}}}{k_1}\,. \tag{S.31}$$

C. Reaction-diffusion equation

In this simulation, we assumed that the molecules diffused through two-dimensional space. The diffusion equation for the molecule is as follows:

$$\frac{\partial u}{\partial t} = D\left(\frac{\partial^2 u}{\partial x^2} + \frac{\partial^2 u}{\partial y^2}\right) = D\nabla^2 u\,, \tag{S.32}$$

where u is the concentration of a molecule, and D is the diffusion coefficient. Considering reaction equations $(\mathrm{S.13})$ to $(\mathrm{S.30})$, the normalized reaction-diffusion equations are represented as follows:

$$\frac{\partial \tilde{u}_{\mathrm{L_{AB}}}}{\partial \tilde{t}} = \tilde{\kappa}\nabla^2 \tilde{u}_{\mathrm{L_{AB}}} - \tilde{k}_{\mathrm{h_{AB}}} \tilde{u}_{\mathrm{L_{AB}}} \tilde{u}_{\mathrm{T_{AB1}}} - \tilde{k}_{\mathrm{h_{AB}}} \tilde{u}_{\mathrm{L_{AB}}} \tilde{u}_{\mathrm{T_{AB2}}}\,, \tag{S.33}$$

$$\frac{\partial \tilde{u}_{\mathrm{T_{AB1}}}}{\partial \tilde{t}} = \tilde{\kappa}\nabla^2 \tilde{u}_{\mathrm{T_{AB1}}} - \tilde{k}_{\mathrm{h_{AB}}} \tilde{u}_{\mathrm{L_{AB}}} \tilde{u}_{\mathrm{T_{AB1}}} - \tilde{k}_{\mathrm{SD_{AB}}} \tilde{u}_{\mathrm{ImtL_{AB2}}} \tilde{u}_{\mathrm{T_{AB1}}}\,, \tag{S.34}$$

$$\frac{\partial \tilde{u}_{\mathrm{T_{AB2}}}}{\partial \tilde{t}} = \tilde{\kappa}\nabla^2 \tilde{u}_{\mathrm{T_{AB2}}} - \tilde{k}_{\mathrm{h_{AB}}} \tilde{u}_{\mathrm{L_{AB}}} \tilde{u}_{\mathrm{T_{AB2}}} - \tilde{k}_{\mathrm{SD_{AB}}} \tilde{u}_{\mathrm{ImtL_{AB1}}} \tilde{u}_{\mathrm{T_{AB2}}}\,, \tag{S.35}$$

$$\frac{\partial \tilde{u}_{\mathrm{ImtL_{AB1}}}}{\partial \tilde{t}} = \tilde{\kappa}\nabla^2 \tilde{u}_{\mathrm{ImtL_{AB1}}} + \tilde{k}_{\mathrm{h_{AB}}} \tilde{u}_{\mathrm{L_{AB}}} \tilde{u}_{\mathrm{T_{AB1}}} - \tilde{k}_{\mathrm{SD_{AB}}} \tilde{u}_{\mathrm{ImtL_{AB1}}} \tilde{u}_{\mathrm{T_{AB2}}}\,, \tag{S.36}$$

$$\frac{\partial \tilde{u}_{\mathrm{ImtL_{AB2}}}}{\partial \tilde{t}} = \tilde{\kappa}\nabla^2 \tilde{u}_{\mathrm{ImtL_{AB2}}} + \tilde{k}_{\mathrm{h_{AB}}} \tilde{u}_{\mathrm{L_{AB}}} \tilde{u}_{\mathrm{T_{AB2}}} - \tilde{k}_{\mathrm{SD_{AB}}} \tilde{u}_{\mathrm{ImtL_{AB2}}} \tilde{u}_{\mathrm{T_{AB1}}}, \tag{S.37}$$

$$\frac{\partial \tilde{u}_{\mathrm{ClvL_{AB1}}}}{\partial \tilde{t}} = \tilde{\kappa}\nabla^2 \tilde{u}_{\mathrm{ClvL_{AB1}}} + \tilde{k}_{\mathrm{SD_{AB}}} \tilde{u}_{\mathrm{ImtL_{AB1}}} \tilde{u}_{\mathrm{T_{AB2}}} + \tilde{k}_{\mathrm{SD_{AB}}} \tilde{u}_{\mathrm{ImtL_{AB2}}} \tilde{u}_{\mathrm{T_{AB1}}}\,, \tag{S.38}$$

$$\frac{\partial \tilde{u}_{\mathrm{ClvL_{AB2}}}}{\partial \tilde{t}} = \tilde{\kappa}\nabla^2 \tilde{u}_{\mathrm{ClvL_{AB2}}} + \tilde{k}_{\mathrm{SD_{AB}}} \tilde{u}_{\mathrm{ImtL_{AB1}}} \tilde{u}_{\mathrm{T_{AB2}}} + \tilde{k}_{\mathrm{SD_{AB}}} \tilde{u}_{\mathrm{ImtL_{AB2}}} \tilde{u}_{\mathrm{T_{AB1}}}\,, \tag{S.39}$$

$$\frac{\partial \tilde{u}_{\mathrm{iT^{\dagger}}_{\mathrm{AB1}}}}{\partial \tilde{t}} = \tilde{\kappa}\nabla^2 \tilde{u}_{\mathrm{iT^{\dagger}}_{\mathrm{AB1}}} - \frac{\tilde{k}_{\mathrm{cat}} \tilde{c}_{\mathrm{E_{RH}}} \tilde{u}_{\mathrm{iT^{\dagger}}_{\mathrm{AB1}}}}{\tilde{K}_{\mathrm{m}} + \tilde{u}_{\mathrm{iT^{\dagger}}_{\mathrm{AB1}}}} + \tilde{k}_{\mathrm{h_{RNA}}} \tilde{u}_{\mathrm{T^{\dagger}}_{\mathrm{AB1}}} \tilde{u}_{\mathrm{R^{\dagger}}_{\mathrm{AB1}}}\,, \tag{S.40}$$

$$\frac{\partial \tilde{u}_{\mathrm{iT^{\dagger}}_{\mathrm{AB2}}}}{\partial \tilde{t}} = \tilde{\kappa}\nabla^2 \tilde{u}_{\mathrm{iT^{\dagger}}_{\mathrm{AB2}}} - \frac{\tilde{k}_{\mathrm{cat}} \tilde{c}_{\mathrm{E_{RH}}} \tilde{u}_{\mathrm{iT^{\dagger}}_{\mathrm{AB2}}}}{\tilde{K}_{\mathrm{m}} + \tilde{u}_{\mathrm{iT^{\dagger}}_{\mathrm{AB2}}}} + \tilde{k}_{\mathrm{h_{RNA}}} \tilde{u}_{\mathrm{T^{\dagger}}_{\mathrm{AB2}}} \tilde{u}_{\mathrm{R^{\dagger}}_{\mathrm{AB2}}}\,, \tag{S.41}$$

$$\frac{\partial \tilde{u}_{\mathrm{R^{\dagger}}_{\mathrm{AB1}}}}{\partial \tilde{t}} = \tilde{\kappa}\nabla^2 \tilde{u}_{\mathrm{R^{\dagger}}_{\mathrm{AB1}}} - \tilde{k}_{\mathrm{h_{RNA}}} \tilde{u}_{\mathrm{T^{\dagger}}_{\mathrm{AB1}}} \tilde{u}_{\mathrm{R^{\dagger}}_{\mathrm{AB1}}}\,, \tag{S.42}$$

$$\frac{\partial \tilde{u}_{\mathrm{R}^\dagger{}_{\mathrm{AB2}}}}{\partial \tilde{t}} = \tilde{\kappa}\nabla^2 \tilde{u}_{\mathrm{R}^\dagger{}_{\mathrm{AB2}}} - \tilde{k}_{\mathrm{h_{RNA}}} \tilde{u}_{\mathrm{T}^\dagger{}_{\mathrm{AB2}}} \tilde{u}_{\mathrm{R}^\dagger{}_{\mathrm{AB2}}} , \tag{S.43}$$

$$\frac{\partial \tilde{u}_{\mathrm{L}^\dagger{}_{\mathrm{AB}}}}{\partial \tilde{t}} = \tilde{\kappa}\nabla^2 \tilde{u}_{\mathrm{L}^\dagger{}_{\mathrm{AB}}} - \tilde{k}_{\mathrm{h_{AB}}} \tilde{u}_{\mathrm{L}^\dagger{}_{\mathrm{AB}}} \tilde{u}_{\mathrm{T}^\dagger{}_{\mathrm{AB1}}} - \tilde{k}_{\mathrm{h_{AB}}} \tilde{u}_{\mathrm{L}^\dagger{}_{\mathrm{AB}}} \tilde{u}_{\mathrm{T}^\dagger{}_{\mathrm{AB2}}} , \tag{S.44}$$

$$\begin{aligned} \frac{\partial \tilde{u}_{\mathrm{T}^\dagger{}_{\mathrm{AB1}}}}{\partial \tilde{t}} &= \tilde{\kappa}\nabla^2 \tilde{u}_{\mathrm{T}^\dagger{}_{\mathrm{AB1}}} + \frac{\tilde{k}_{\mathrm{cat}} \tilde{c}_{\mathrm{E_{RH}}} \tilde{u}_{\mathrm{iT}^\dagger{}_{\mathrm{AB1}}}}{\tilde{K}_{\mathrm{m}} + \tilde{u}_{\mathrm{iT}^\dagger{}_{\mathrm{AB1}}}} - \tilde{k}_{\mathrm{h_{RNA}}} \tilde{u}_{\mathrm{T}^\dagger{}_{\mathrm{AB1}}} \tilde{u}_{\mathrm{R}^\dagger{}_{\mathrm{AB1}}} \\ &\quad - \tilde{k}_{\mathrm{h_{AB}}} \tilde{u}_{\mathrm{L}^\dagger{}_{\mathrm{AB}}} \tilde{u}_{\mathrm{T}^\dagger{}_{\mathrm{AB1}}} - \tilde{k}_{\mathrm{SD_{AB}}} \tilde{u}_{\mathrm{ImtL}^\dagger{}_{\mathrm{AB2}}} \tilde{u}_{\mathrm{T}^\dagger{}_{\mathrm{AB1}}} , \end{aligned} \tag{S.45}$$

$$\begin{aligned} \frac{\partial \tilde{u}_{\mathrm{T}^\dagger{}_{\mathrm{AB2}}}}{\partial \tilde{t}} &= \tilde{\kappa}\nabla^2 \tilde{u}_{\mathrm{T}^\dagger{}_{\mathrm{AB2}}} + \frac{\tilde{k}_{\mathrm{cat}} \tilde{c}_{\mathrm{E_{RH}}} \tilde{u}_{\mathrm{iT}^\dagger{}_{\mathrm{AB2}}}}{\tilde{K}_{\mathrm{m}} + \tilde{u}_{\mathrm{iT}^\dagger{}_{\mathrm{AB2}}}} - \tilde{k}_{\mathrm{h_{RNA}}} \tilde{u}_{\mathrm{T}^\dagger{}_{\mathrm{AB2}}} \tilde{u}_{\mathrm{R}^\dagger{}_{\mathrm{AB2}}} \\ &\quad - \tilde{k}_{\mathrm{h_{AB}}} \tilde{u}_{\mathrm{L}^\dagger{}_{\mathrm{AB}}} \tilde{u}_{\mathrm{T}^\dagger{}_{\mathrm{AB2}}} - \tilde{k}_{\mathrm{SD_{AB}}} \tilde{u}_{\mathrm{ImtL}^\dagger{}_{\mathrm{AB1}}} \tilde{u}_{\mathrm{T}^\dagger{}_{\mathrm{AB2}}} , \end{aligned} \tag{S.46}$$

$$\frac{\partial \tilde{u}_{\mathrm{ImtL}^\dagger{}_{\mathrm{AB1}}}}{\partial \tilde{t}} = \tilde{\kappa}\nabla^2 \tilde{u}_{\mathrm{ImtL}^\dagger{}_{\mathrm{AB1}}} + \tilde{k}_{\mathrm{h_{AB}}} \tilde{u}_{\mathrm{L}^\dagger{}_{\mathrm{AB}}} \tilde{u}_{\mathrm{T}^\dagger{}_{\mathrm{AB1}}} - \tilde{k}_{\mathrm{SD_{AB}}} \tilde{u}_{\mathrm{ImtL}^\dagger{}_{\mathrm{AB1}}} \tilde{u}_{\mathrm{T}^\dagger{}_{\mathrm{AB2}}} , \tag{S.47}$$

$$\frac{\partial \tilde{u}_{\mathrm{ImtL}^\dagger{}_{\mathrm{AB2}}}}{\partial \tilde{t}} = \tilde{\kappa}\nabla^2 \tilde{u}_{\mathrm{ImtL}^\dagger{}_{\mathrm{AB2}}} + \tilde{k}_{\mathrm{h_{AB}}} \tilde{u}_{\mathrm{L}^\dagger{}_{\mathrm{AB}}} \tilde{u}_{\mathrm{T}^\dagger{}_{\mathrm{AB2}}} - \tilde{k}_{\mathrm{SD_{AB}}} \tilde{u}_{\mathrm{ImtL}^\dagger{}_{\mathrm{AB2}}} \tilde{u}_{\mathrm{T}^\dagger{}_{\mathrm{AB1}}} , \tag{S.48}$$

$$\frac{\partial \tilde{u}_{\mathrm{ClvL}^\dagger{}_{\mathrm{AB1}}}}{\partial \tilde{t}} = \tilde{\kappa}\nabla^2 \tilde{u}_{\mathrm{ClvL}^\dagger{}_{\mathrm{AB1}}} + \tilde{k}_{\mathrm{SD_{AB}}} \tilde{u}_{\mathrm{ImtL}^\dagger{}_{\mathrm{AB1}}} \tilde{u}_{\mathrm{T}^\dagger{}_{\mathrm{AB2}}} + \tilde{k}_{\mathrm{SD_{AB}}} \tilde{u}_{\mathrm{ImtL}^\dagger{}_{\mathrm{AB2}}} \tilde{u}_{\mathrm{T}^\dagger{}_{\mathrm{AB1}}} , \tag{S.49}$$

$$\frac{\partial \tilde{u}_{\mathrm{ClvL}^\dagger{}_{\mathrm{AB2}}}}{\partial \tilde{t}} = \tilde{\kappa}\nabla^2 \tilde{u}_{\mathrm{ClvL}^\dagger{}_{\mathrm{AB2}}} + \tilde{k}_{\mathrm{SD_{AB}}} \tilde{u}_{\mathrm{ImtL}^\dagger{}_{\mathrm{AB1}}} \tilde{u}_{\mathrm{T}^\dagger{}_{\mathrm{AB2}}} + \tilde{k}_{\mathrm{SD_{AB}}} \tilde{u}_{\mathrm{ImtL}^\dagger{}_{\mathrm{AB2}}} \tilde{u}_{\mathrm{T}^\dagger{}_{\mathrm{AB1}}} , \tag{S.50}$$

for which the concentrations, lengths, time, and other parameters were normalized as follows:

$$\tilde{u}_{\mathrm{X}} = \frac{u_{\mathrm{X}}}{u^0_{\mathrm{L_{total}}}} , \tag{S.51}$$

$$\tilde{c}_{\mathrm{E_{RH}}} = \frac{c_{\mathrm{E_{RH}}}}{u^0_{\mathrm{L_{total}}}} , \tag{S.52}$$

$$\tilde{t} = k_{\mathrm{h_{RNA}}} u^0_{\mathrm{L_{total}}} t , \tag{S.52}$$

$$\tilde{x} = \frac{x}{l_0} , \tag{S.53}$$

$$\tilde{y} = \frac{y}{l_0} , \tag{S.54}$$

$$\tilde{\kappa} = \frac{D}{k_{\mathrm{h_{RNA}}} u^0_{\mathrm{L_{total}}} l_0^2} , \tag{S.55}$$

$$\tilde{k}_{\mathrm{h_{RNA}}} = \frac{k_{\mathrm{h_{RNA}}}}{k_{\mathrm{h_{RNA}}}} , \tag{S.56}$$

$$\tilde{k}_{\mathrm{h_{AB}}} = \frac{k_{\mathrm{h_{AB}}}}{k_{\mathrm{h_{RNA}}}} , \tag{S.57}$$

$$\tilde{k}_{cat} = \frac{k_{cat}}{k_{\mathrm{h_{RNA}}} u^0_{\mathrm{L_{total}}}} , \quad (\mathrm{S.58})$$

$$\tilde{k}_{\mathrm{SD_{AB}}} = \frac{k_{\mathrm{SD_{AB}}}}{k_{\mathrm{h_{RNA}}}} , \quad (\mathrm{S.59})$$

$$\tilde{K}_m = \frac{K_m}{u^0_{\mathrm{L_{total}}}} . \quad (\mathrm{S.60})$$

Here, u_{X} is the molecule 'X' concentration. $u^0_{\mathrm{L_{total}}}$ is the total initial concentration of the DNA linkers and is defined as

$$u^0_{\mathrm{L_{total}}} = u^0_{\mathrm{L_{AB}}} + u^0_{\mathrm{L^{\dagger}{}_{AB}}} , \quad (\mathrm{S.61})$$

where $u^0_{\mathrm{L_{AB}}}$ and $u^0_{\mathrm{L^{\dagger}{}_{AB}}}$ are the initial concentration of L_{AB} and $L^{\dagger}_{AB}$, respectively. l_0 is the characteristic length.

D. Numerical Methods

We discretized the reaction-diffusion equation as follows:

$$\frac{\tilde{u}^{k+1}_{\mathrm{X}}(x_i, y_j) - \tilde{u}^{k}_{\mathrm{X}}(x_i, y_j)}{\Delta\tilde{t}} = \frac{\tilde{\kappa}}{\Delta\tilde{h}^2}\Big(\tilde{u}^{k}_{\mathrm{X}}(x_{i+1}, y_j) + \tilde{u}^{k}_{\mathrm{X}}(x_{i-1}, y_j) + \tilde{u}^{k}_{\mathrm{X}}(x_i, y_{j+1}) + \tilde{u}^{k}_{\mathrm{X}}(x_i, y_{j-1}) - 4\tilde{u}^{k}_{\mathrm{X}}(x_i, y_j)\Big) + v(\dots) \quad (\mathrm{S.62})$$

where $\tilde{u}^{k}_{\mathrm{X}}(x_i, y_j)$ is the concentration of molecule 'X' at time k and position (x_i, y_j). $v(\dots)$ is the reaction term. $\Delta\tilde{h}$ is the grid spacing. We set up a two-dimensional space with a side length of L as the entire reaction space. We assumed that a mixed DNA droplet of radius R existed at the center of the space. An equidistant discretization of 100 points along each dimension was performed, and $\Delta\tilde{h}$ was defined as $\Delta\tilde{h} = \tilde{L}/100,$ where $\tilde{L} = L/l_0$ We imposed the periodic boundary condition as the boundary condition of the entire reaction space. We assumed that the DNA linker molecules always existed within the droplet area, whereas the other molecules initially existed outside the droplet area and gradually invaded it. The diffusion coefficients inside and outside the droplet were set to D_{in} and D_{out}, respectively.

Based on these equations, we performed numerical simulations using Python 3.7. The reaction-diffusion equations were solved explicitly using the fourth-order Runge-Kutta Method. We referred to the kinetic parameter values reported in other papers: $K_{\mathrm{m}} = 3.9 \times 10^{-1}$ [μM][1]; $k_{\mathrm{cat}} = 8.2 \times 10^{-2}$ [s^{-1}][1]; $k_{\mathrm{h_{RNA}}} = k_{\mathrm{h_{AB}}} = 10$ [μM^{-1}s^{-1}][2]; and $k_{\mathrm{SD_{AB}}} =$

$6.0\ [\mu M^{-1}s^{-1}]$[3]. The initial concentration of each component is set as follows: $u^0_{L_{AB1}} = 3.713 \times 10^{-1}\ [\mu M]$; $u^0_{T_{AB1}} = u^0_{T_{AB2}} = 1.25\ [\mu M]$; $u^0_{ImtL_{AB1}} = u^0_{ImtL_{AB2}} = u^0_{ClvL_{AB1}} = u^0_{ClvL_{AB2}} = 0\ [\mu M]$; $u^0_{L^\dagger{}_{AB1}} = 4.125 \times 10^{-2}\ [\mu M]$; $u^0_{T^\dagger{}_{AB1}} = u^0_{T^\dagger{}_{AB2}} = 0\ [\mu M]$; $u^0_{iT^\dagger{}_{AB1}} = u^0_{iT^\dagger{}_{AB2}} = 1.25 \times 10^{-1}\ [\mu M]$; $u^0_{ImtL^\dagger{}_{AB1}} = u^0_{ImtL^\dagger{}_{AB2}} = u^0_{ClvL^\dagger{}_{AB1}} = u^0_{ClvL^\dagger{}_{AB2}} = 0\ [\mu M]$. The initial concentration of $R^\dagger{}_{AB1}$ and $R^\dagger{}_{AB2}$ ($u^0_{R^\dagger{}_{AB1}}$ and $u^0_{R^\dagger{}_{AB2}}$) was varied: $0, 6.25 \times 10^{-2}$, and $1.25 \times 10^{-1}\ [\mu M]$. The normalized initial total concentration of inhibitor RNA $\tilde{c}_{AB} = u^{tot}_{R^\dagger{}_{ABi}}/u^{tot}_{T^\dagger{}_{ABi}} = (u^0_{R^\dagger{}_{ABi}} + u^0_{iT^\dagger{}_{ABi}})/(u^0_{T^\dagger{}_{ABi}} + u^0_{iT^\dagger{}_{ABi}})$ is defined. $c_{E_{RH}}$ is also varied: $4.25 \times 10^{-3}, 8.5 \times 10^{-3}$ and $1.7 \times 10^{-2}\ [\mu M]$, which correspond to experimental enzyme unit concentrations $1.25 \times 10^{-2}, 2.5 \times 10^{-2}$, and $5.0 \times 10^{-2}\ [U/\mu L]$[2]. In the simulation, we defined the length parameters as follows: $L = 50\ [\mu m]$, $l_0 = 0.5\ [\mu m]$, and $R = 15\ [\mu m]$. We set the diffusion coefficient outside the droplet $D_{out} = 6.22\ [\mu m^2/s]$[4]. Considering the effect of a viscous environment in the DNA droplet on the diffusiveness of DNA molecules, the diffusion coefficient inside the droplet was set as $D_{in} = 0.35\ [\mu m^2/s]$ based on previous research[5]. For simplicity, we neglected the difference in the diffusion coefficients with respect to the length of the DNA. In a previous study, we observed a sigmoidal curve for the division ratio of a mixed DNA droplet[6]; thus, we assumed a cooperative model for the relationship between the division ratio and concentration of uncleaved linker DNAs. In this simulation, we defined the division ratio of a mixed DNA droplet r_{div} using the Hill function as follows:

$$r_{div} = \frac{H(w) - H_{min}}{H_{max} - H_{min}}\,, \qquad (S.63)$$

$$H(w) = \frac{K^n}{K^n + w^n}\,, \qquad (S.64)$$

$$w = \tilde{u}_{L_{AB}} + \tilde{u}_{ImtL_{AB1}} + \tilde{u}_{ImtL_{AB2}} + \tilde{u}_{L^\dagger{}_{AB}} + \tilde{u}_{ImtL^\dagger{}_{AB1}} + \tilde{u}_{ImtL^\dagger{}_{AB2}}\,, \qquad (S.65)$$

where K is the threshold concentration of the uncleaved linker for A:B-droplet division and n is a cooperativity coefficient to express the switch-like dependence of division on the uncleaved linker concentration w. H_{max} and H_{min} are the maximum and minimum values of $H(w)$, respectively. n was varied between 2, 4, 8, 16, 32. K was varied between 0.01, 0.05, 0.10.

Supplementary Note 2: Reaction-diffusion simulation of Linker cleavage reactions of $L^{\dagger}_{AB}$ and $L^{\dagger}_{AC}$

A. Reaction-diffusion equation

Reaction-diffusion equations of the time-delay circuit and the cleavage reaction for $L^{\dagger}_{AB}$ are $(\mathrm{S.}40)$~$(\mathrm{S.}50)$. Chemical reactions for the time-delay circuit and cleavage reaction of $L^{\dagger}_{AC}$ are expressed in the same way as the chemical reactions for $L^{\dagger}_{AB}$. The reactions are as follows:

$$\mathrm{T^{\dagger}}_{\mathrm{AC1}} + \mathrm{R^{\dagger}}_{\mathrm{AC1}} \overset{k_{\mathrm{hRNA}}}{\longrightarrow} \mathrm{iT^{\dagger}}_{\mathrm{AC1}}\,, \tag{S.66}$$

$$\mathrm{T^{\dagger}}_{\mathrm{AC2}} + \mathrm{R^{\dagger}}_{\mathrm{AC2}} \overset{k_{\mathrm{hRNA}}}{\longrightarrow} \mathrm{iT^{\dagger}}_{\mathrm{AC2}}\,, \tag{S.67}$$

$$\mathrm{iT^{\dagger}}_{\mathrm{AC1}} + \mathrm{E_{RH}} \underset{k_2}{\overset{k_1}{\rightleftarrows}} \mathrm{iT^{\dagger}}_{\mathrm{AC1}}\mathrm{E_{RH}} \overset{k_{\mathrm{cat}}}{\longrightarrow} \mathrm{T^{\dagger}}_{\mathrm{AC1}} + \mathrm{E_{RH}}\,, \tag{S.68}$$

$$\mathrm{iT^{\dagger}}_{\mathrm{AC2}} + \mathrm{E_{RH}} \underset{k_2}{\overset{k_1}{\rightleftarrows}} \mathrm{iT^{\dagger}}_{\mathrm{AC2}}\mathrm{E_{RH}} \overset{k_{\mathrm{cat}}}{\longrightarrow} \mathrm{T^{\dagger}}_{\mathrm{AC2}} + \mathrm{E_{RH}}\,, \tag{S.69}$$

$$\mathrm{L^{\dagger}}_{\mathrm{AC}} + \mathrm{T^{\dagger}}_{\mathrm{AC1}} \overset{k_{\mathrm{h_{AC}}}}{\longrightarrow} \mathrm{ItmL^{\dagger}}_{\mathrm{AC1}}\,, \tag{S.70}$$

$$\mathrm{L^{\dagger}}_{\mathrm{AC}} + \mathrm{T^{\dagger}}_{\mathrm{AC2}} \overset{k_{\mathrm{h_{AC}}}}{\longrightarrow} \mathrm{ItmL^{\dagger}}_{\mathrm{AC2}}\,, \tag{S.71}$$

$$\mathrm{ItmL^{\dagger}}_{\mathrm{AC1}} + \mathrm{T^{\dagger}}_{\mathrm{AC2}} \overset{k_{\mathrm{SD_{AC}}}}{\longrightarrow} \mathrm{ClvL^{\dagger}}_{\mathrm{AC1}} + \mathrm{ClvL^{\dagger}}_{\mathrm{AC2}}\,, \tag{S.72}$$

$$\mathrm{ItmL^{\dagger}}_{\mathrm{AC2}} + \mathrm{T^{\dagger}}_{\mathrm{AC1}} \overset{k_{\mathrm{SD_{AC}}}}{\longrightarrow} \mathrm{ClvL^{\dagger}}_{\mathrm{AC1}} + \mathrm{ClvL^{\dagger}}_{\mathrm{AC2}}\,. \tag{S.73}$$

where $\mathrm{iT^{\dagger}}_{\mathrm{AC1}}\mathrm{E_{RH}}$ and $\mathrm{iT^{\dagger}}_{\mathrm{AC2}}\mathrm{E_{RH}}$ are the enzyme-substrate complexes; $k_{\mathrm{h_{AC}}}$ is the rate constant of the hybridization between division triggers and $L^{\dagger}_{AC}$; $k_{\mathrm{SD_{AC}}}$ is the rate constant of the strand displacement of $L^{\dagger}_{AC}$.

Based on these chemical reaction equations, the normalized reaction-diffusion equations for the time-delay circuit and cleavage reactions of $L^{\dagger}_{AC}$ are described as follows:

$$\frac{\partial \tilde{u}_{\mathrm{iT^{\dagger}}_{\mathrm{AC1}}}}{\partial \tilde{t}} = \tilde{\kappa}\nabla^2 \tilde{u}_{\mathrm{iT^{\dagger}}_{\mathrm{AC1}}} - \frac{\tilde{k}_{\mathrm{cat}}\tilde{c}_{\mathrm{E_{RH}}}\tilde{u}_{\mathrm{iT^{\dagger}}_{\mathrm{AC1}}}}{\tilde{K}_{\mathrm{m}} + \tilde{u}_{\mathrm{iT^{\dagger}}_{\mathrm{AC1}}}} + \tilde{k}_{\mathrm{hRNA}}\tilde{u}_{\mathrm{T^{\dagger}}_{\mathrm{AC1}}}\tilde{u}_{\mathrm{R^{\dagger}}_{\mathrm{AC1}}}\,, \tag{S.74}$$

$$\frac{\partial \tilde{u}_{\mathrm{iT^{\dagger}}_{\mathrm{AC2}}}}{\partial \tilde{t}} = \tilde{\kappa}\nabla^2 \tilde{u}_{\mathrm{iT^{\dagger}}_{\mathrm{AC2}}} - \frac{\tilde{k}_{\mathrm{cat}}\tilde{c}_{\mathrm{E_{RH}}}\tilde{u}_{\mathrm{iT^{\dagger}}_{\mathrm{AC2}}}}{\tilde{K}_{\mathrm{m}} + \tilde{u}_{\mathrm{iT^{\dagger}}_{\mathrm{AC2}}}} + \tilde{k}_{\mathrm{hRNA}}\tilde{u}_{\mathrm{T^{\dagger}}_{\mathrm{AC2}}}\tilde{u}_{\mathrm{R^{\dagger}}_{\mathrm{AC2}}}\,, \tag{S.75}$$

$$\frac{\partial \tilde{u}_{\mathrm{R^\dagger}_{\mathrm{AC1}}}}{\partial \tilde{t}} = \tilde{\kappa}\nabla^2 \tilde{u}_{\mathrm{R^\dagger}_{\mathrm{AC1}}} - \tilde{k}_{\mathrm{hRNA}} \tilde{u}_{\mathrm{T^\dagger}_{\mathrm{AC1}}} \tilde{u}_{\mathrm{R^\dagger}_{\mathrm{AC1}}}, \tag{S.76}$$

$$\frac{\partial \tilde{u}_{\mathrm{R^\dagger}_{\mathrm{AC2}}}}{\partial \tilde{t}} = \tilde{\kappa}\nabla^2 \tilde{u}_{\mathrm{R^\dagger}_{\mathrm{AC2}}} - \tilde{k}_{\mathrm{hRNA}} \tilde{u}_{\mathrm{T^\dagger}_{\mathrm{AC2}}} \tilde{u}_{\mathrm{R^\dagger}_{\mathrm{AC2}}}, \tag{S.77}$$

$$\frac{\partial \tilde{u}_{\mathrm{L^\dagger}_{\mathrm{AC}}}}{\partial \tilde{t}} = \tilde{\kappa}\nabla^2 \tilde{u}_{\mathrm{L^\dagger}_{\mathrm{AC}}} - \tilde{k}_{\mathrm{h_{AC}}} \tilde{u}_{\mathrm{L^\dagger}_{\mathrm{AC}}} \tilde{u}_{\mathrm{T^\dagger}_{\mathrm{AC1}}} - \tilde{k}_{\mathrm{h_{AC}}} \tilde{u}_{\mathrm{L^\dagger}_{\mathrm{AC}}} \tilde{u}_{\mathrm{T^\dagger}_{\mathrm{AC2}}}, \tag{S.78}$$

$$\frac{\partial \tilde{u}_{\mathrm{T^\dagger}_{\mathrm{AC1}}}}{\partial \tilde{t}} = \tilde{\kappa}\nabla^2 \tilde{u}_{\mathrm{T^\dagger}_{\mathrm{AC1}}} + \frac{\tilde{k}_{\mathrm{cat}} \tilde{c}_{\mathrm{E_{RH}}} \tilde{u}_{\mathrm{iT^\dagger}_{\mathrm{AC1}}}}{\tilde{K}_{\mathrm{m}} + \tilde{u}_{\mathrm{iT^\dagger}_{\mathrm{AC1}}}} - \tilde{k}_{\mathrm{hRNA}} \tilde{u}_{\mathrm{T^\dagger}_{\mathrm{AC1}}} \tilde{u}_{\mathrm{R^\dagger}_{\mathrm{AC1}}}$$
$$-\tilde{k}_{\mathrm{h_{AC}}} \tilde{u}_{\mathrm{L^\dagger}_{\mathrm{AC}}} \tilde{u}_{\mathrm{T^\dagger}_{\mathrm{AC1}}} - \tilde{k}_{\mathrm{SD_{AC}}} \tilde{u}_{\mathrm{ImtL^\dagger}_{\mathrm{AC2}}} \tilde{u}_{\mathrm{T^\dagger}_{\mathrm{AC1}}}, \tag{S.79}$$

$$\frac{\partial \tilde{u}_{\mathrm{T^\dagger}_{\mathrm{AC2}}}}{\partial \tilde{t}} = \tilde{\kappa}\nabla^2 \tilde{u}_{\mathrm{T^\dagger}_{\mathrm{AC2}}} + \frac{\tilde{k}_{\mathrm{cat}} \tilde{c}_{\mathrm{E_{RH}}} \tilde{u}_{\mathrm{iT^\dagger}_{\mathrm{AC2}}}}{\tilde{K}_{\mathrm{m}} + \tilde{u}_{\mathrm{iT^\dagger}_{\mathrm{AC2}}}} - \tilde{k}_{\mathrm{hRNA}} \tilde{u}_{\mathrm{T^\dagger}_{\mathrm{AC2}}} \tilde{u}_{\mathrm{R^\dagger}_{\mathrm{AC2}}}$$
$$-\tilde{k}_{\mathrm{h_{AC}}} \tilde{u}_{\mathrm{L^\dagger}_{\mathrm{AC}}} \tilde{u}_{\mathrm{T^\dagger}_{\mathrm{AC2}}} - \tilde{k}_{\mathrm{SD_{AC}}} \tilde{u}_{\mathrm{ImtL^\dagger}_{\mathrm{AC1}}} \tilde{u}_{\mathrm{T^\dagger}_{\mathrm{AC2}}}, \tag{S.80}$$

$$\frac{\partial \tilde{u}_{\mathrm{ImtL^\dagger}_{\mathrm{AC1}}}}{\partial \tilde{t}} = \tilde{\kappa}\nabla^2 \tilde{u}_{\mathrm{ImtL^\dagger}_{\mathrm{AC1}}} + \tilde{k}_{\mathrm{h_{AC}}} \tilde{u}_{\mathrm{L^\dagger}_{\mathrm{AC}}} \tilde{u}_{\mathrm{T^\dagger}_{\mathrm{AC1}}} - \tilde{k}_{\mathrm{SD_{AC}}} \tilde{u}_{\mathrm{ImtL^\dagger}_{\mathrm{AC1}}} \tilde{u}_{\mathrm{T^\dagger}_{\mathrm{AC2}}}, \tag{S.81}$$

$$\frac{\partial \tilde{u}_{\mathrm{ImtL^\dagger}_{\mathrm{AC2}}}}{\partial \tilde{t}} = \tilde{\kappa}\nabla^2 \tilde{u}_{\mathrm{ImtL^\dagger}_{\mathrm{AC2}}} + \tilde{k}_{\mathrm{h_{AC}}} \tilde{u}_{\mathrm{L^\dagger}_{\mathrm{AC}}} \tilde{u}_{\mathrm{T^\dagger}_{\mathrm{AC2}}} - \tilde{k}_{\mathrm{SD_{AC}}} \tilde{u}_{\mathrm{ImtL^\dagger}_{\mathrm{AC2}}} \tilde{u}_{\mathrm{T^\dagger}_{\mathrm{AC1}}}, \tag{S.82}$$

$$\frac{\partial \tilde{u}_{\mathrm{ClvL^\dagger}_{\mathrm{AC1}}}}{\partial \tilde{t}} = \tilde{\kappa}\nabla^2 \tilde{u}_{\mathrm{ClvL^\dagger}_{\mathrm{AC1}}} + \tilde{k}_{\mathrm{SD_{AC}}} \tilde{u}_{\mathrm{ImtL^\dagger}_{\mathrm{AC1}}} \tilde{u}_{\mathrm{T^\dagger}_{\mathrm{AC2}}} + \tilde{k}_{\mathrm{SD_{AC}}} \tilde{u}_{\mathrm{ImtL^\dagger}_{\mathrm{AC2}}} \tilde{u}_{\mathrm{T^\dagger}_{\mathrm{AC1}}}, \tag{S.83}$$

$$\frac{\partial \tilde{u}_{\mathrm{ClvL^\dagger}_{\mathrm{AC2}}}}{\partial \tilde{t}} = \tilde{\kappa}\nabla^2 \tilde{u}_{\mathrm{ClvL^\dagger}_{\mathrm{AC2}}} + \tilde{k}_{\mathrm{SD_{AC}}} \tilde{u}_{\mathrm{ImtL^\dagger}_{\mathrm{AC1}}} \tilde{u}_{\mathrm{T^\dagger}_{\mathrm{AC2}}} + \tilde{k}_{\mathrm{SD_{AC}}} \tilde{u}_{\mathrm{ImtL^\dagger}_{\mathrm{AC2}}} \tilde{u}_{\mathrm{T^\dagger}_{\mathrm{AC1}}}. \tag{S.84}$$

We normalized the concentrations, lengths, times, and other parameters similarly to $(S.51)$~$(S.60)$. Here, we defined

$$u^0_{\mathrm{L_{total}}} = u^0_{\mathrm{L^\dagger}_{\mathrm{AB}}} = u^0_{\mathrm{L^\dagger}_{\mathrm{AC}}}, \tag{S.85}$$

where $u^0_{\mathrm{L^\dagger}_{\mathrm{AB}}}$ and $u^0_{\mathrm{L^\dagger}_{\mathrm{AC}}}$ are the initial concentration of $L^\dagger_{AB}$ and $L^\dagger_{AC}$, respectively.

C. Numerical Methods

The numerical method used for the simulation was the same as that described in Supplementary Note 1 D. We used the kinetic parameter values reported in other papers: $K_{\mathrm{m}} = 3.9 \times 10^{-1}\ [\mu\mathrm{M}]$; $k_{\mathrm{cat}} = 8.2 \times 10^{-2}\ [\mathrm{s}^{-1}]$; $k_{\mathrm{h_{RNA}}} = k_{\mathrm{h_{AC}}} = 10\ [\mu\mathrm{M}^{-1}\mathrm{s}^{-1}]$; and $k_{\mathrm{SD_{AC}}} = 6.0\ [\mu\mathrm{M}^{-1}\mathrm{s}^{-1}]$. $k_{\mathrm{h_{AB}}}$ was varied as $1.0 \times 10^{-1}, 1.0, 10\ [\mu\mathrm{M}^{-1}\mathrm{s}^{-1}]$. $k_{\mathrm{SD_{AB}}}$ was also varied as $6.0 \times 10^{-2}, 6.0 \times 10^{-1}, 6.0\ [\mu\mathrm{M}^{-1}\mathrm{s}^{-1}]$. The initial concentrations of triggers and inhibitor RNAs varied, as shown in Supplementary Table 11. The normalized initial total concentration of inhibitor RNA $\tilde{c}_{\mathrm{AB}} = u^{\mathrm{tot}}_{\mathrm{R^\dagger}_{\mathrm{AB}i}} / u^{\mathrm{tot}}_{\mathrm{T^\dagger}_{\mathrm{AB}i}} = (u^0_{\mathrm{R^\dagger}_{\mathrm{AB}i}} + u^0_{\mathrm{iT^\dagger}_{\mathrm{AB}i}})/(u^0_{\mathrm{T^\dagger}_{\mathrm{AB}i}} + u^0_{\mathrm{iT^\dagger}_{\mathrm{AB}i}})$, where $u^{\mathrm{tot}}_{\mathrm{R^\dagger}_{\mathrm{AB}i}} = u^0_{\mathrm{R^\dagger}_{\mathrm{AB}i}} + u^0_{\mathrm{iT^\dagger}_{\mathrm{AB}i}}$ is the initial total concentration of excess and hybridized inhibitor RNAs, and $u^{\mathrm{tot}}_{\mathrm{T^\dagger}_{\mathrm{AB}i}} = u^0_{\mathrm{T^\dagger}_{\mathrm{AB}i}} + u^0_{\mathrm{iT^\dagger}_{\mathrm{AB}i}}$ is the initial total concentration of active and inhibited triggers. Similarly, $\tilde{c}_{\mathrm{AC}} = u^{\mathrm{tot}}_{\mathrm{R^\dagger}_{\mathrm{AC}i}} / u^{\mathrm{tot}}_{\mathrm{T^\dagger}_{\mathrm{AC}i}} = (u^0_{\mathrm{R^\dagger}_{\mathrm{AC}i}} + u^0_{\mathrm{iT^\dagger}_{\mathrm{AC}i}})/(u^0_{\mathrm{T^\dagger}_{\mathrm{AC}i}} + u^0_{\mathrm{iT^\dagger}_{\mathrm{AC}i}})$. The initial concentrations of DNA linker particles are fixed as follows; $u^0_{\mathrm{L^\dagger}_{\mathrm{AB}}} = u^0_{\mathrm{L^\dagger}_{\mathrm{AC}}} = 8.0 \times 10^{-1}\ [\mu\mathrm{M}]$; $u^0_{\mathrm{ImtL^\dagger}_{\mathrm{AB1}}} = u^0_{\mathrm{ImtL^\dagger}_{\mathrm{AB2}}} = u^0_{\mathrm{ClvL^\dagger}_{\mathrm{AB1}}} = u^0_{\mathrm{ClvL^\dagger}_{\mathrm{AB2}}} = u^0_{\mathrm{ImtL^\dagger}_{\mathrm{AC1}}} = u^0_{\mathrm{ImtL^\dagger}_{\mathrm{AC2}}} = u^0_{\mathrm{ClvL^\dagger}_{\mathrm{AC1}}} = u^0_{\mathrm{ClvL^\dagger}_{\mathrm{AC2}}} = 0\ [\mu\mathrm{M}]$. $c_{\mathrm{E_{RH}}}$ is fixed at $8.5 \times 10^{-2}\ [\mu\mathrm{M}]$, which correspond to experimental enzyme unit concentrations $2.5 \times 10^{-1}\ [\mathrm{U}/\mu\mathrm{L}]$. The length parameters are set as follows: $L = 125\ [\mu\mathrm{m}]$, $l_0 = 1.25\ [\mu\mathrm{m}]$, and $R = 18.75\ [\mu\mathrm{m}]$. D_{out} and D_{in} was set as 6.22 and $0.35\ [\mu\mathrm{m}^2/\mathrm{s}]$, respectively. For simplicity, we neglected the difference in the diffusion coefficients with respect to the length of DNA. We defined the division ratio of B-droplets, $r_{\mathrm{div\_B}}$, and C-droplets, $r_{\mathrm{div\_C}}$, as follows:

$$w_{\mathrm{AB}} = \tilde{u}_{\mathrm{L^\dagger}_{\mathrm{AB}}} + \tilde{u}_{\mathrm{ImtL^\dagger}_{\mathrm{AB1}}} + \tilde{u}_{\mathrm{ImtL^\dagger}_{\mathrm{AB2}}}\ , \tag{S.86}$$

$$w_{\mathrm{AC}} = \tilde{u}_{\mathrm{L^\dagger}_{\mathrm{AC}}} + \tilde{u}_{\mathrm{ImtL^\dagger}_{\mathrm{AC1}}} + \tilde{u}_{\mathrm{ImtL^\dagger}_{\mathrm{AC2}}}\ , \tag{S.87}$$

$$f(w_{\mathrm{AB}}) = \frac{K^n_{\mathrm{AB}}}{K^n_{\mathrm{AB}} + w^n_{\mathrm{AB}}}\ , \tag{S.88}$$

$$g(w_{\mathrm{AC}}) = \frac{K^n_{\mathrm{AC}}}{K^n_{\mathrm{AC}} + w^n_{\mathrm{AC}}}\ , \tag{S.89}$$

$$r_{\mathrm{div\_B}} = \frac{f(w_{\mathrm{AB}}) - f_{\mathrm{min}}}{f_{\mathrm{max}} - f_{\mathrm{min}}}\ , \tag{S.90}$$

$$r_{\mathrm{div\_C}} = \frac{g(w_{\mathrm{AC}}) - g_{\mathrm{min}}}{g_{\mathrm{max}} - g_{\mathrm{min}}}\ , \tag{S.91}$$

where K_{AB} and K_{AC} are the threshold concentrations of the uncleaved linker for A·B-droplet division and A·C-droplet division, respectively. n is a cooperativity coefficient that expresses the switch-like dependence of division on the uncleaved linker concentrations. f_{max} and f_{min} are the maximum and minimum values of $f(w_{\mathrm{AB}})$, respectively. g_{max} and g_{min} denote the maximum and minimum value of $g(w_{\mathrm{AC}})$,

respectively. n was varied as 2, 4, 8, 16, 32. K_{AB} and K_{AC} were varied as 0.1, 0.5, and 0.9.

Supplementary Note 3: Measurement of a division ratio in the experiment

The fluorescence images in the 6-FAM, Alexa405, and Cy3 channels were converted to binary images using Fiji[7], and the droplet regions were distinguished from other regions. The division ratio for B-droplet division, $r_{\mathrm{div\_B}}$, was defined as follows: $r_{\mathrm{div\_B}} = 1 - N_{\mathrm{colc\_AB}}/N_{\mathrm{total\_B}}$, where $N_{\mathrm{total\_B}}$ is the sum of the number of pixels in the droplet regions in the Alexa 405 channel; $N_{\mathrm{colc\_AB}}$ is the sum of the number of pixels that are droplet regions in both the 6-FAM channel and the Alexa405 channel. In addition, the division ratio for C-droplet division, $r_{\mathrm{div\_C}}$, was defined as follows: $r_{\mathrm{div\_C}} = 1 - N_{\mathrm{colc\_AC}}/N_{\mathrm{total\_C}}$, where $N_{\mathrm{total\_C}}$ is the sum of the number of pixels in the droplet regions in the Cy3 channel and $N_{\mathrm{colc\_AC}}$ is the sum of the number of pixels that are droplet regions in both the 6-FAM and Cy3 channels. We measured the time course of $r_{\mathrm{div\_B}}$ in Figures 4c and 4d and that of both $r_{\mathrm{div\_B}}$ and $r_{\mathrm{div\_C}}$ in Figure 7a.

Supplementary Figures

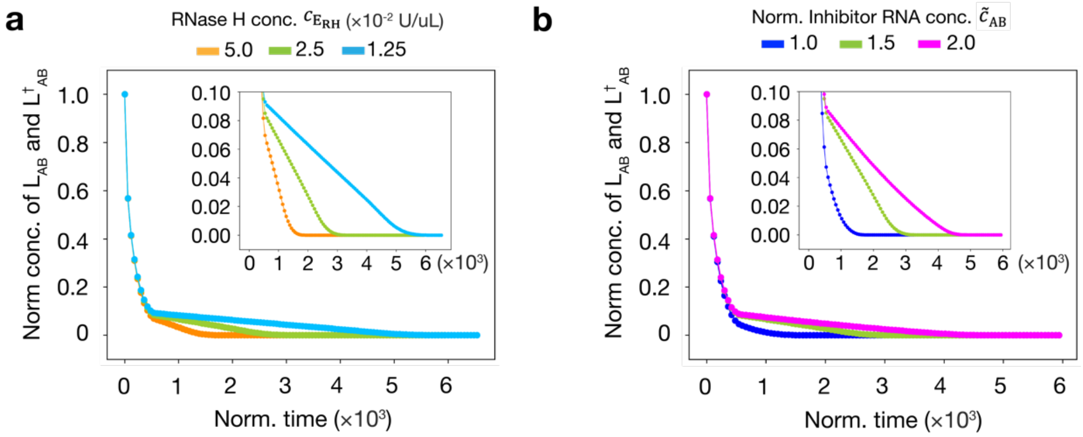

Supplementary Figure 1. Time course of total DNA linker concentration in the reaction-diffusion simulation (**a**) Upon decreasing RNase H concentration, $c_{\mathrm{E_{RH}}}$, the cleavage rate of total DNA linker gets more slowly. $\tilde{c}_{\mathrm{AB}}$ was fixed at 1.5. (**b**) Upon increasing Inhibitor RNA concentrations, $u^0_{\mathrm{R^{\dagger}}_{\mathrm{AB1}}}$ and $u^0_{\mathrm{R^{\dagger}}_{\mathrm{AB2}}}$, the cleavage rate of total DNA linker is slowed. $c_{\mathrm{E_{RH}}}$ was fixed at 2.5×10^{-2} U/μL. These results suggested that the cleavage rate of total DNA linker can be regulated by tuning $c_{\mathrm{E_{RH}}}$ or $u^0_{\mathrm{R^{\dagger}}_{\mathrm{AB}i}}$ (i =1, 2). Source data are provided as a Source Data file.

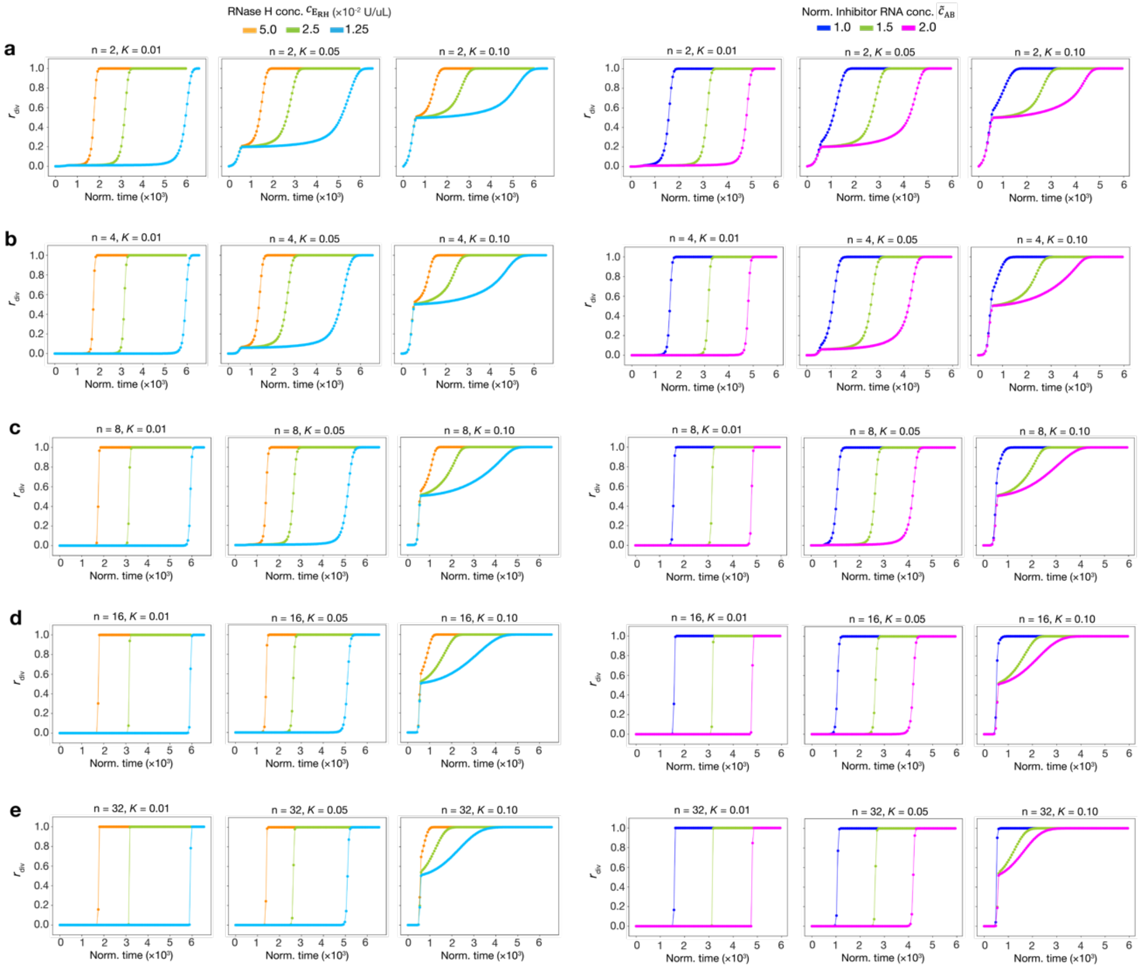

Supplementary Figure 2. Time course of the division ratio r_{div} in the reaction-diffusion simulation. Time courses of r_{div} at n = 2 (**a**), 4 (**b**), 8 (**c**), 16 (**d**), and 32 (**e**). For each condition, the value of K was changed to 0.01, 0.05, and 0.10. In all results, we consistently observed the trend of the increasing rate of r_{div} become slow when increasing $u^0_{R^\dagger{}_{ABi}}$ (i =1, 2) or decreasing $c_{E_{RH}}$. Source data are provided as a Source Data file.

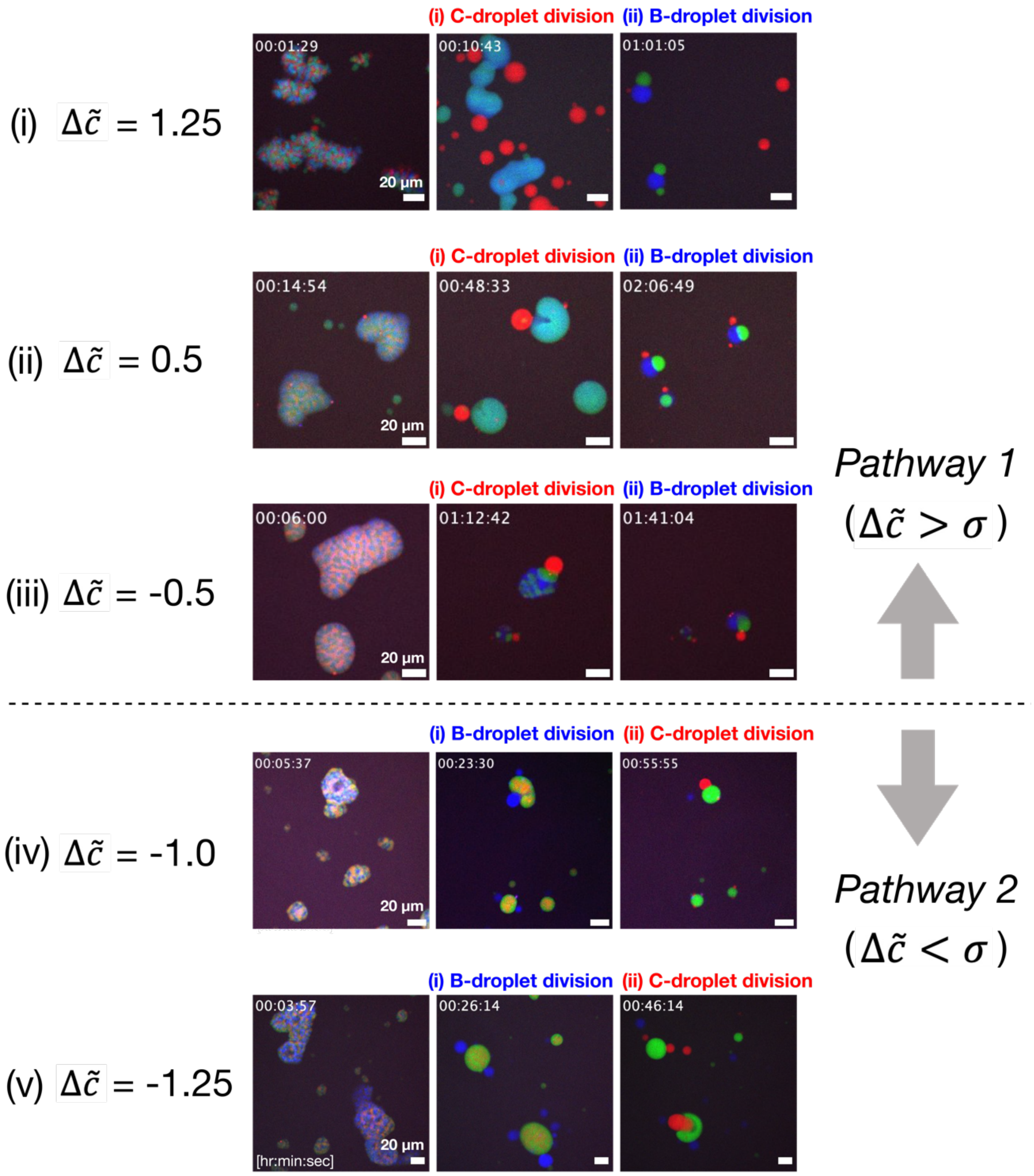

Supplementary Figure 3. Time-lapse images of C·A·B-droplet division at several RNA conditions (i)-(v). More than four independent fields of view were observed with similar results.

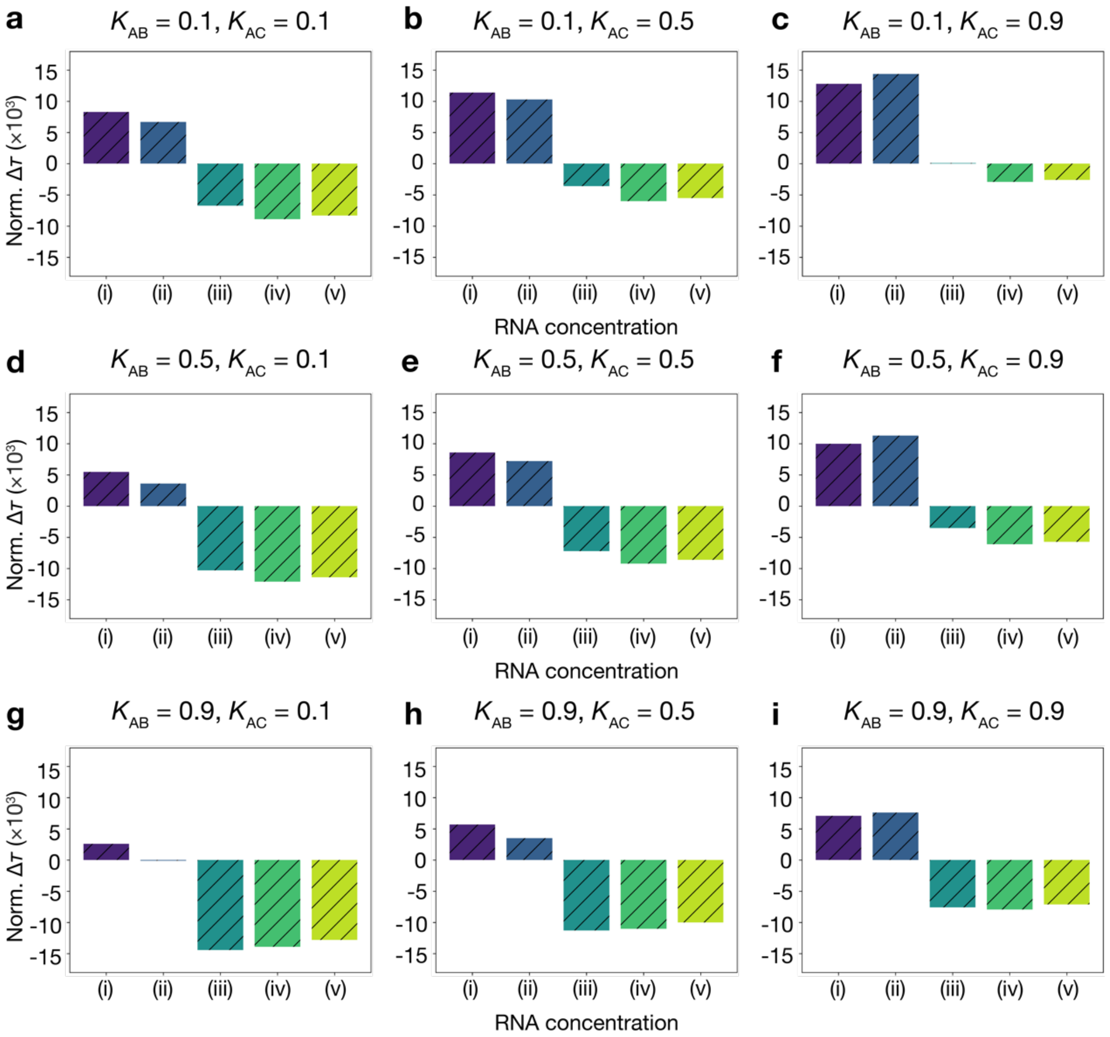

Supplementary Figure 4. Time difference $\Delta\tau$ at each of five RNA conditions in the reaction-diffusion simulation when $\boldsymbol{k_{h_{AB}}/k_{h_{AC}} = k_{SD_{AB}}/k_{SD_{AC}} = 1.0}$. $\Delta\tilde{c} =$ (i) 1.25, (ii) 0.5, (iii) -0.5, (iv) -1.0, and (v) -1.25. *n* = 16. Source data are provided as a Source Data file.

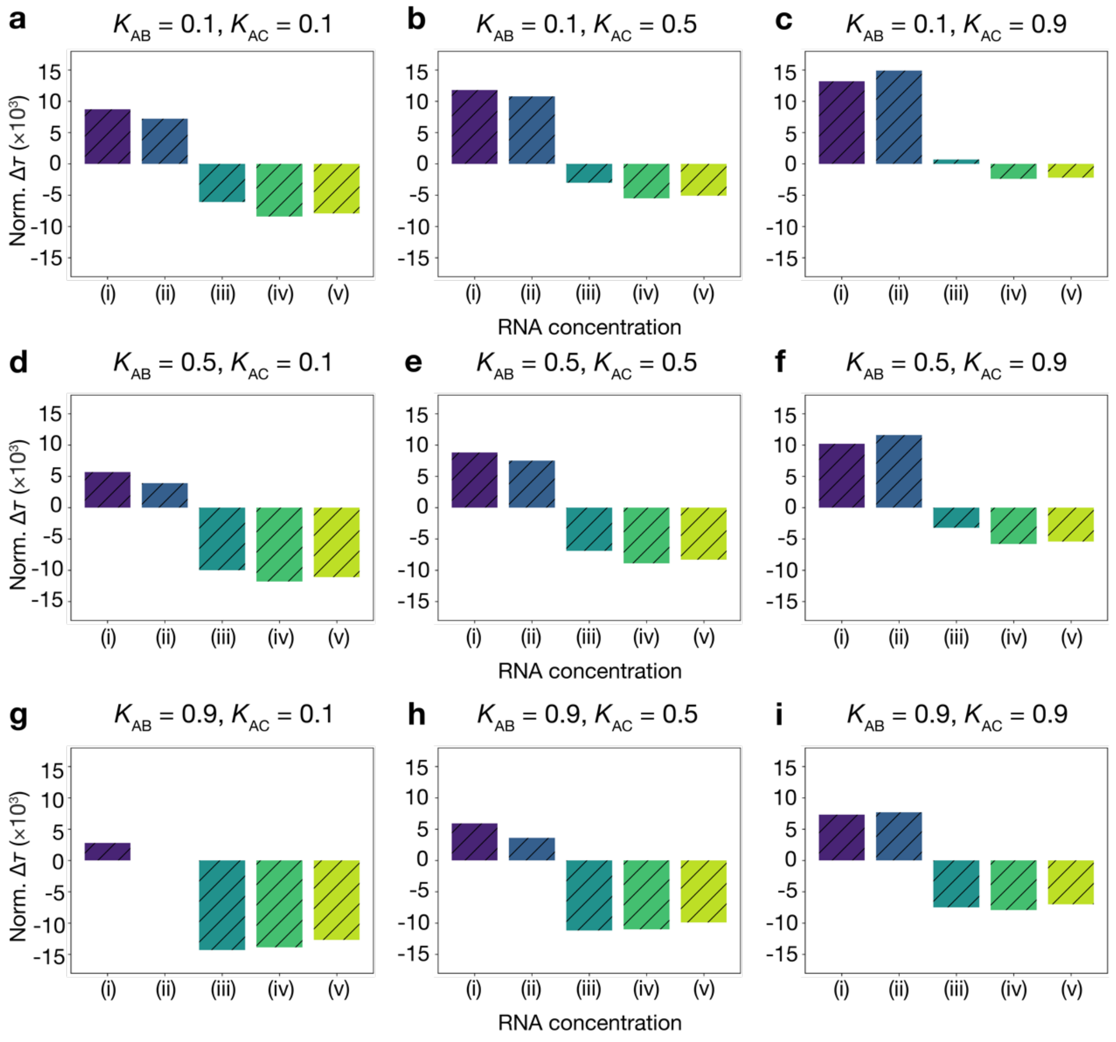

Supplementary Figure 5. Time difference $\Delta\tau$ at each of five RNA conditions in the reaction-diffusion simulation when $k_{h_{AB}}/k_{h_{AC}} = k_{SD_{AB}}/k_{SD_{AC}} = 0.1$. $\Delta\tilde{c} =$ (i) 1.25, (ii) 0.5, (iii) -0.5, (iv) -1.0, and (v) -1.25. *n* = 16. Source data are provided as a Source Data file.

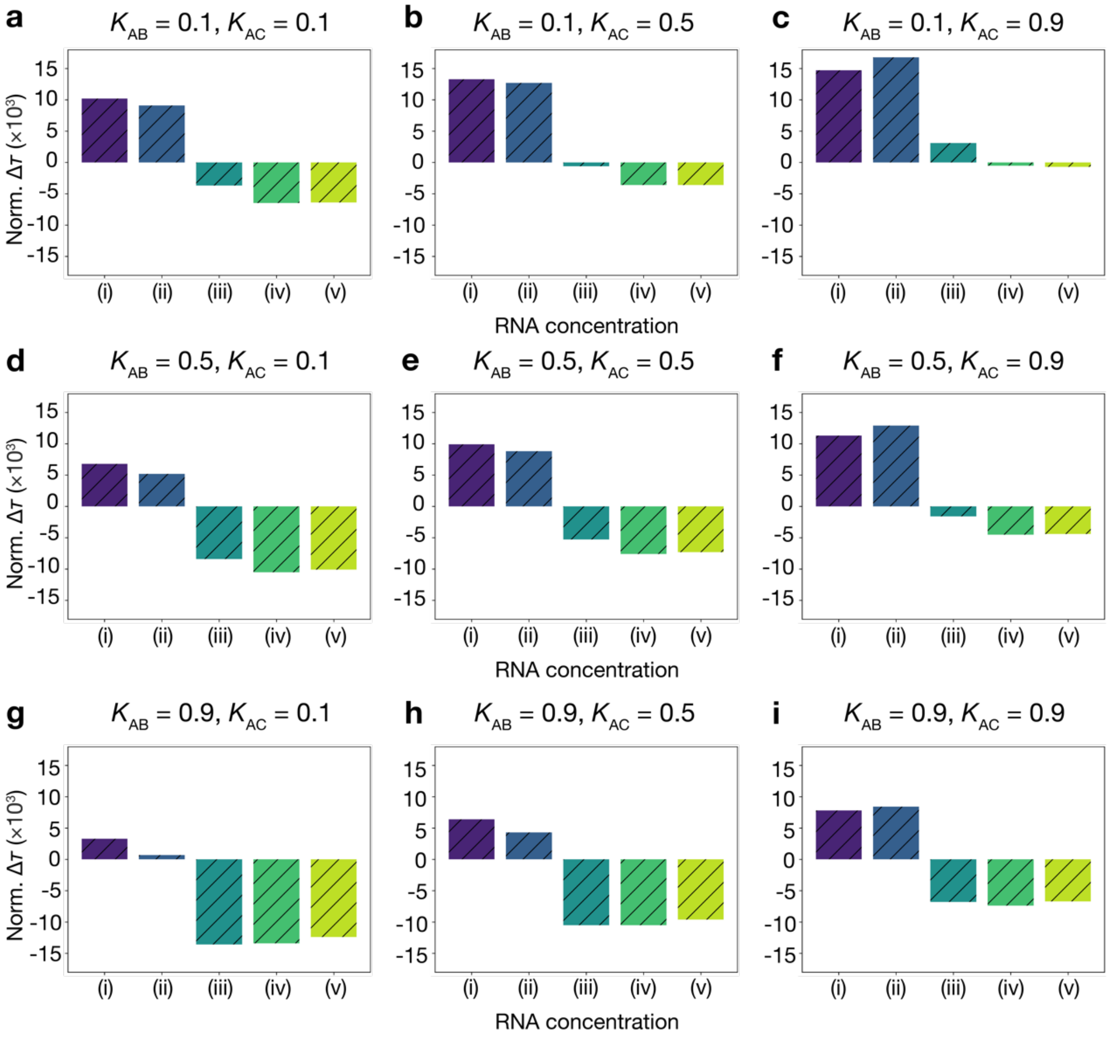

Supplementary Figure 6. Time difference $\Delta\tau$ at each of five RNA conditions in the reaction-diffusion simulation when $k_{h_{AB}}/k_{h_{AC}} = k_{SD_{AB}}/k_{SD_{AC}} = 0.01$. $\Delta\tilde{c} =$ (i) 1.25, (ii) 0.5, (iii) -0.5, (iv) -1.0, and (v) -1.25. n = 16. Source data are provided as a Source Data file.

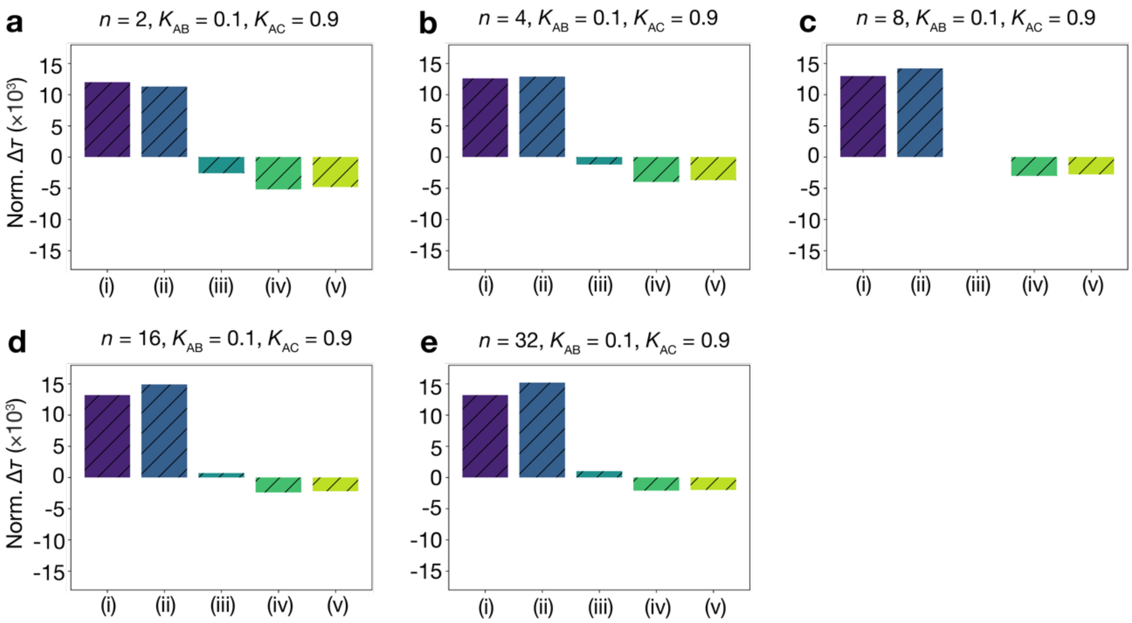

Supplementary Figure 7. Time difference $\Delta\tau$ at each of five RNA conditions in the reaction-diffusion simulation. $\Delta\tilde{c} =$ (i) 1.25, (ii) 0.5, (iii) -0.5, (iv) -1.0, and (v) -1.25. $k_{h_{AB}}/k_{h_{AC}} = k_{SD_{AB}}/k_{SD_{AC}} = 0.1$ and n = 2 (a), 4 (b), 8 (c), 16 (d), and 32 (e). Source data are provided as a Source Data file.

Supplementary Tables

Supplementary Table 1: Sequences of Y-shaped DNA nanostructures. Bold font represents the sticky end sequence. Complementary stem sequences are represented in the same color.

Name	Sequence (5'→3')
Y_{A1}	**GCTCGAGC**CAGTGAGGACGGAAGTTTGTCGTAGCATCGCACC
Y_{A2}	**GCTCGAGC**CAACCACGCCTGTCCATTACTTCCGTCCTCACTG
Y_{A3}	**GCTCGAGC**GGTGCGATGCTACGACTTTGGACAGGCGTGGTTG
Y_{A2}_0_FAM	**[FAM]-**CAACCACGCCTGTCCATTACTTCCGTCCTCACTG
Y_{B1}	**CTCGCGAG**AAAGGAACTCTCCGCGTTGACAAAGCCGACACGT
Y_{B2}	**CTCGCGAG**GCCTCTGTGTCGCATCTTCGCGGAGAGTTCCTTT
Y_{B3}	**CTCGCGAG**ACGTGTCGGCTTTGTCTTGATGCGACACAGAGGC
Y_{B2}_0_Alexa405	**[Alexa405]-**GCCTCTGTGTCGCATCTTCGCGGAGAGTTCCTTT
Y_{C1}	**CAGCGCTG**CTGGTTACACTGAGCTTTATGAACCTAGTGTGGC
Y_{C2}	**CAGCGCTG**GCCACACTAGGTTCATTTCGCTTGATACGATGTC
Y_{C3}	**CAGCGCTG**GACATCGTATCAAGCGTTAGCTCAGTGTAACCAG
Y_{C2}_0_Cy3	**[Cy3]-**GCCACACTAGGTTCATTTCGCTTGATACGATGTC

Supplementary Table 2: Sequences of 6-branched DNA linkers. Bold font represents the sticky end sequence. The underlined sequence represents the Toehold sequences. Complementary stem sequences are represented in the same color.

Name	Sequence (5'→3')
L_{AB1}	**GCTCGAGC**CACGACCGACGCCACGCCGAGTTTGGTGGCTTATACAGACGT
L_{AB2}	**GCTCGAGC**ACGTCTGTATAAGCCACCTTTCGGTTCTCTCCAAAGCA
L_{AB3}	**GCTCGAGC**TGCTTTGGAGAGAACCGATTAATGGATTTTTGGA
L_{AB4}	**CTCGCGAG**CCTGCTCCAAAAATCCATTTTTGCGAAATTGATGGCTGC
L_{AB5}	**CTCGCGAG**GCAGCCATCAATTTCGCATTCGGTCACATAACTGGAGA
L_{AB6}	**CTCGCGAG**TCTCCAGTTATGTGACCGTTACTCGGCGTGG
$L^{\dagger}_{AB1}$	**GCTCGAGC**CGGCGCTGTAAATTTGCGTTCCCCGGGCCGGT
$L^{\dagger}_{AB2}$	**GCTCGAGC**CAGACGTCACTCTCCAACTTCGCAAATTTACAGCGCCG
$L^{\dagger}_{AB3}$	**GCTCGAGC**TGAGGGACCCAGGACAGGAGATTGTTGGAGAGTGACGTCTG
$L^{\dagger}_{AB4}$	**CTCGCGAG**GCTGGACTAACGGAACGGTTTCTCCTGTCCTGG
$L^{\dagger}_{AB5}$	**CTCGCGAG**CTCAGAGAGGTGACAGCATTCCGTTCCGTTAGTCCAGC
$L^{\dagger}_{AB6}$	**CTCGCGAG**CGGCGCGACCGGCCCGGGGTTTGCTGTCACCTCTCTGAG
$L^{\dagger}_{AC1}$	**GCTCGAGC**AATGGATTTTTGGAGCAGGTTGGTGGCTTATACAGACGT
$L^{\dagger}_{AC2}$	**GCTCGAGC**ACGTCTGTATAAGCCACCTTTCGGTTCTCTCCAAAGCA
$L^{\dagger}_{AC3}$	**GCTCGAGC**TGCTTTGGAGAGAACCGATTCACGACCGACG
$L^{\dagger}_{AC4}$	**CAGCGCTG**ACTCGGCGTGGCGTCGGTCGTGTTTGCGAAATTGATGGCTGC
$L^{\dagger}_{AC5}$	**CAGCGCTG**GCAGCCATCAATTTCGCATTCGGTCACATAACTGGAGA
$L^{\dagger}_{AC6}$	**CAGCGCTG**TCTCCAGTTATGTGACCGTTCCTGCTCCAAAAA

Supplementary Table 3: Sequences of division triggers and Inhibitor RNAs. T_{AB1}, T_{AB2}, $T^{\dagger}_{AB1}$, $T^{\dagger}_{AB2}$, $T^{\dagger}_{AC1}$, and $T^{\dagger}_{AC2}$ are DNA sequences. $R^{\dagger}_{AB1}$, $R^{\dagger}_{AB2}$, $R^{\dagger}_{AC1}$, and $R^{\dagger}_{AC2}$ are RNA sequences. The underlined sequence represents the Toehold sequences. Here, the sequences of $R^{\dagger}_{AB1}$, $R^{\dagger}_{AB2}$, $R^{\dagger}_{AC1}$, and $R^{\dagger}_{AC2}$ are the same as miR-6875-5p, miR-4634, miR-1246, and miR-1307-3p, respectively.

Name	Sequence (5'→3')
T_{AB1}	AATGGATTTTTGGA<u>GCAGG</u>
T_{AB2}	ACTCGGCGTGG<u>CGTCGGTCGTG</u>
$T^{\dagger}_{AB1}$	TCTCCTGTCCTGG<u>GTCCCTCA</u>
$T^{\dagger}_{AB2}$	CCCCGGGCCGGT<u>CGCGCCG</u>
$R^{\dagger}_{AB1}$	UGAGGGACCCAGGACAGGAGA
$R^{\dagger}_{AB2}$	CGGCGCGACCGGCCCGGGG
$T^{\dagger}_{AC1}$	CCTGCTCCAAAAA<u>TCCATT</u>
$T^{\dagger}_{AC2}$	CACGACCGACG<u>CCACGCCGAGT</u>
$R^{\dagger}_{AC1}$	AAUGGAUUUUUGGAGCAGG
$R^{\dagger}_{AC2}$	ACUCGGCGUGGCGUCGGUCGUG

Supplementary Table 4: Strand concentrations of A·B-droplet

Name	Concentration		
	At annealing	After dilution	After adding the mixture
Y_{A1}	5.0 µM	2.5 µM	1.25 µM
Y_{A2}	4.5 µM	2.25 µM	1.125 µM
Y_{A3}	5.0 µM	2.5 µM	1.25 µM
Y_{A2}_0_FAM	0.5 µM	0.25 µM	0.125 µM
Y_{B1}	5.0 µM	2.5 µM	1.25 µM
Y_{B2}	4.5 µM	2.25 µM	1.125 µM
Y_{B3}	5.0 µM	2.5 µM	1.25 µM
Y_{B2}_0_Alexa405	0.5 µM	0.25 µM	0.125 µM
L_{AB1}	1.65 µM	0.825 µM	0.4125 µM
L_{AB2}	1.65 µM	0.825 µM	0.4125 µM
L_{AB3}	1.65 µM	0.825 µM	0.4125 µM
L_{AB4}	1.65 µM	0.825 µM	0.4125 µM
L_{AB5}	1.65 µM	0.825 µM	0.4125 µM
L_{AB6}	1.65 µM	0.825 µM	0.4125 µM

Supplementary Table 5: Strand concentrations of A:B-droplet

Name	Concentration		
	**At annealing**	**After dilution**	**After adding the mixture**
Y_{A1}	5.0 µM	2.5 µM	1.25 µM
Y_{A2}	4.5 µM	2.25 µM	1.125 µM
Y_{A3}	5.0 µM	2.5 µM	1.25 µM
Y_{A2}_0_FAM	0.5 µM	0.25 µM	0.125 µM
Y_{B1}	5.0 µM	2.5 µM	1.25 µM
Y_{B2}	4.5 µM	2.25 µM	1.125 µM
Y_{B3}	5.0 µM	2.5 µM	1.25 µM
Y_{B2}_0_Alexa405	0.5 µM	0.25 µM	0.125 µM
L_{AB1}	1.485 µM	0.7425 µM	0.37125 µM
L_{AB2}	1.485 µM	0.7425 µM	0.37125 µM
L_{AB3}	1.485 µM	0.7425 µM	0.37125 µM
L_{AB4}	1.485 µM	0.7425 µM	0.37125 µM
L_{AB5}	1.485 µM	0.7425 µM	0.37125 µM
L_{AB6}	1.485 µM	0.7425 µM	0.37125 µM
$L^{\dagger}_{AB1}$	0.165 µM	0.0825 µM	0.04125 µM
$L^{\dagger}_{AB2}$	0.165 µM	0.0825 µM	0.04125 µM
$L^{\dagger}_{AB3}$	0.165 µM	0.0825 µM	0.04125 µM
$L^{\dagger}_{AB4}$	0.165 µM	0.0825 µM	0.04125 µM
$L^{\dagger}_{AB5}$	0.165 µM	0.0825 µM	0.04125 µM
$L^{\dagger}_{AB6}$	0.165 µM	0.0825 µM	0.04125 µM

Supplementary Table 6: Strand concentrations of C·A·B-droplet

Name	Concentration	
	**At annealing**	**After adding the mixture**
Y_{A1}	1.0 µM	0.4 µM
Y_{A2}	0.9 µM	0.36 µM
Y_{A3}	1.0 µM	0.4 µM
Y_{A2}_0_FAM	0.1 µM	0.04 µM
Y_{B1}	1.0 µM	0.4 µM
Y_{B2}	0.9 µM	0.36 µM
Y_{B3}	1.0 µM	0.4 µM
Y_{B2}_0_Alexa405	0.1 µM	0.04 µM
Y_{C1}	1.0 µM	0.4 µM
Y_{C2}	0.9 µM	0.36 µM
Y_{C3}	1.0 µM	0.4 µM
Y_{C2}_0_Cy3	0.1 µM	0.04 µM
$L^{\dagger}_{AB1}$	2.0 µM	0.8 µM
$L^{\dagger}_{AB2}$	2.0 µM	0.8 µM
$L^{\dagger}_{AB3}$	2.0 µM	0.8 µM
$L^{\dagger}_{AB4}$	2.0 µM	0.8 µM
$L^{\dagger}_{AB5}$	2.0 µM	0.8 µM
$L^{\dagger}_{AB6}$	2.0 µM	0.8 µM
$L^{\dagger}_{AC1}$	2.0 µM	0.8 µM
$L^{\dagger}_{AC2}$	2.0 µM	0.8 µM
$L^{\dagger}_{AC3}$	2.0 µM	0.8 µM

$L^{\dagger}_{AC4}$	2.0 μM	0.8 μM
$L^{\dagger}_{AC5}$	2.0 μM	0.8 μM
$L^{\dagger}_{AC6}$	2.0 μM	0.8 μM

Supplementary Table 7: Strand and enzyme concentrations in a trigger mixture for the timing-controlled division experiment

Name	Concentration	
	**Before mixing with the droplet sample**	**After mixing with the droplet sample**
T_{AB1}	2.5 µM	1.25 µM
T_{AB2}	2.5 µM	1.25 µM
$T^{\dagger}_{AB1}$	0.25 µM	0.125 µM
$T^{\dagger}_{AB2}$	0.25 µM	0.125 µM
$R^{\dagger}_{AB1}$	0.25 / 0.375 / 0.5 µM	0.125 / 0.1875 / 0.25 µM
$R^{\dagger}_{AB2}$	0.25 / 0.375 / 0.5 µM	0.125 / 0.1875 / 0.25 µM
Thermostable RNase H	0.025 / 0.05 / 0.1 U/µL	0.0125 / 0.025 / 0.05 U/µL
$MgCl_2$	3 mM	1.5 mM

Supplementary Table 8: Strand and enzyme concentrations in a trigger mixture 1 for the division through Pathway 1

Name	Concentration	
	Before mixing with the droplet sample	After mixing with the droplet sample
$T^{\dagger}_{AB1}$	4 µM	2.4 µM
$T^{\dagger}_{AB2}$	4 µM	2.4 µM
$R^{\dagger}_{AB1}$	5 µM	3 µM
$R^{\dagger}_{AB2}$	5 µM	3 µM
$T^{\dagger}_{AC1}$	4 µM	2.4 µM
$T^{\dagger}_{AC2}$	4 µM	2.4 µM
RNase H	0.417 U/µL	0.25 U/µL
$MgCl_2$	2.5 mM	1.5 mM

Supplementary Table 9: Strand and enzyme concentrations in a trigger mixture 2 for the division through Pathway 2

Name	Concentration	
	Before mixing with the droplet sample	After mixing with the droplet sample
$T^{\dagger}_{AB1}$	4 μM	2.4 μM
$T^{\dagger}_{AB2}$	4 μM	2.4 μM
$T^{\dagger}_{AC1}$	4 μM	2.4 μM
$T^{\dagger}_{AC2}$	4 μM	2.4 μM
$R^{\dagger}_{AC1}$	5 μM	3 μM
$R^{\dagger}_{AC2}$	5 μM	3 μM
RNase H	0.417 U/μL	0.25 U/μL
$MgCl_2$	2.5 mM	1.5 mM

Supplementary Table 10: Strand and enzyme concentrations of trigger mixture for the experiment of miRNA sequences concentration comparator

	Concentration after mixing with the droplet sample				
**Name**	**(1)**<br>$\tilde{c}_{AB}$ = 1.25<br>$\tilde{c}_{AC}$ = 0<br>$\Delta\tilde{c}$ = 1.25<br>$\tilde{c}_{t}$ = 1.25	**(2)**<br>$\tilde{c}_{AB}$ = 1.25<br>$\tilde{c}_{AC}$ = 0.75<br>$\Delta\tilde{c}$ = 0.50<br>$\tilde{c}_{t}$ = 2.00	**(3)**<br>$\tilde{c}_{AB}$ = 0.75<br>$\tilde{c}_{AC}$ = 1.25<br>$\Delta\tilde{c}$ = −0.50<br>$\tilde{c}_{t}$ = 2.00	**(4)**<br>$\tilde{c}_{AB}$ = 0.25<br>$\tilde{c}_{AC}$ = 1.25<br>$\Delta\tilde{c}$ = −1.00<br>$\tilde{c}_{t}$ = 1.50	**(5)**<br>$\tilde{c}_{AB}$ = 0<br>$\tilde{c}_{AC}$ = 1.25<br>$\Delta\tilde{c}$ = −1.25<br>$\tilde{c}_{t}$ = 1.25
$T^{\dagger}_{AB1}$	2.4 µM	2.4 µM	2.4 µM	2.4 µM	2.4 µM
$T^{\dagger}_{AB2}$	2.4 µM	2.4 µM	2.4 µM	2.4 µM	2.4 µM
$T^{\dagger}_{AC1}$	2.4 µM	2.4 µM	2.4 µM	2.4 µM	2.4 µM
$T^{\dagger}_{AC2}$	2.4 µM	2.4 µM	2.4 µM	2.4 µM	2.4 µM
$R^{\dagger}_{AB1}$	3 µM	3 µM	2.25 µM	0.75 µM	0 µM
$R^{\dagger}_{AB2}$	3 µM	3 µM	2.25 µM	0.75 µM	0 µM
$R^{\dagger}_{AC1}$	0 µM	2.25 µM	3 µM	3 µM	3 µM
$R^{\dagger}_{AC2}$	0 µM	2.25 µM	3 µM	3 µM	3 µM
RNase H	0.25 U/µL	0.25 U/µL	0.25 U/µL	0.25 U/µL	0.25 U/µL
$MgCl_2$	1.5 mM	1.5 mM	1.5 mM	1.5 mM	1.5 mM

Supplementary Table 11: Concentrations of division triggers and inhibitor RNAs for the reaction-diffusion simulation

Name	Concentration				
	(1)<br>$\tilde{c}_{AB}$ = 1.25<br>$\tilde{c}_{AC}$ = 0<br>$\Delta\tilde{c}$ = 1.25<br>$\tilde{c}_{t}$ = 1.25	(2)<br>$\tilde{c}_{AB}$ = 1.25<br>$\tilde{c}_{AC}$ = 0.75<br>$\Delta\tilde{c}$ = 0.50<br>$\tilde{c}_{t}$ = 2.00	(3)<br>$\tilde{c}_{AB}$ = 0.75<br>$\tilde{c}_{AC}$ = 1.25<br>$\Delta\tilde{c}$ = −0.50<br>$\tilde{c}_{t}$ = 2.00	(4)<br>$\tilde{c}_{AB}$ = 0.25<br>$\tilde{c}_{AC}$ = 1.25<br>$\Delta\tilde{c}$ = −1.00<br>$\tilde{c}_{t}$ = 1.50	(5)<br>$\tilde{c}_{AB}$ = 0<br>$\tilde{c}_{AC}$ = 1.25<br>$\Delta\tilde{c}$ = −1.25<br>$\tilde{c}_{t}$ = 1.25
$T^{\dagger}_{AB1}$	2.4 µM	2.4 µM	2.4 µM	2.4 µM	2.4 µM
$T^{\dagger}_{AB2}$	2.4 µM	2.4 µM	2.4 µM	2.4 µM	2.4 µM
$T^{\dagger}_{AC1}$	2.4 µM	2.4 µM	2.4 µM	2.4 µM	2.4 µM
$T^{\dagger}_{AC2}$	2.4 µM	2.4 µM	2.4 µM	2.4 µM	2.4 µM
$R^{\dagger}_{AB1}$	3 µM	3 µM	2.25 µM	0.75 µM	0 µM
$R^{\dagger}_{AB2}$	3 µM	3 µM	2.25 µM	0.75 µM	0 µM
$R^{\dagger}_{AC1}$	0 µM	2.25 µM	3 µM	3 µM	3 µM
$R^{\dagger}_{AC2}$	0 µM	2.25 µM	3 µM	3 µM	3 µM

References

1. Hirano, N., Haruki, M., Morikawa, M. & Kanaya, S. Enhancement of the enzymatic activity of ribonuclease HI from Thermus thermophilus HB8 with a suppressor mutation method. *Biochemistry* **39**, 13285–13294 (2000).
2. Takinoue, M., Kiga, D., Shohda, K.-I. & Suyama, A. Experiments and simulation models of a basic computation element of an autonomous molecular computing system. *Phys. Rev. E* **78**, 041921 (2008).
3. Zhang, D. Y. & Winfree, E. Control of DNA strand displacement kinetics using toehold exchange. *J. Am. Chem. Soc.* **131**, 17303–17314 (2009).
4. Lukacs, G. L. *et al.* Size-dependent DNA mobility in cytoplasm and nucleus. *J. Biol. Chem.* **275**, 1625–1629 (2000).
5. Sato, Y. & Takinoue, M. Sequence-dependent fusion dynamics and physical properties of DNA droplets. *Nanoscale Adv.* **5**, 1919–1925 (2023).
6. Gong, J., Tsumura, N., Sato, Y. & Takinoue, M. Computational DNA droplets recognizing miRNA sequence inputs based on liquid–liquid phase separation. *Adv. Funct. Mater.* 2202322 (2022).
7. Schindelin, J. *et al.* Fiji: an open-source platform for biological-image analysis. *Nat. Methods* **9**, 676–682 (2012).
