# Supplementary material for: Temporally controlled multistep division of DNA droplets for dynamic artificial cells
Source: Nat Commun. 2024 Aug 27;15:7397. doi: 10.1038/s41467-024-51299-5 (PMC11350047; doi:10.1038/s41467-024-51299-5)
Supplement: Supplementary file 3 — Description of Additional Supplementary Files [file 41467_2024_51299_MOESM3_ESM.pdf]

# Description of Additional Supplementary Files

**Supplementary Movie 1:** Timing-controlled division of A:Bdroplet at $c$ERH = 1.25 × 10−2 U/µL and $c$A$\tilde{B}$ = 1.5.

**Supplementary Movie 2:** Timing-controlled division of A:Bdroplet at $c$ERH = 2.5 × 10−2 U/µL and $c$A$\tilde{B}$ = 1.5.

**Supplementary Movie 3:** Timing-controlled division of A:Bdroplet at $c$ERH = 5.0 × 10−2 U/µL and $c$A$\tilde{B}$ = 1.5.

**Supplementary Movie 4:** Timing-controlled division of A:Bdroplet at $c$ERH = 2.5 × 10−2 U/µL and $c$A$\tilde{B}$ = 1.0.

**Supplementary Movie 5:** Timing-controlled division of A:Bdroplet at $c$ERH = 2.5 × 10−2 U/µL and $c$A$\tilde{B}$ = 2.0.

**Supplementary Movie 6:** Pathway-controlled division of C·A·B-droplets via Pathway 1 (RNA concentration condition (i) in Figure 7a).

**Supplementary Movie 7:** Pathway-controlled division of C·A·B-droplets via Pathway 2 (RNA concentration condition (v) in Figure 7a).

**Supplementary Movie 8:** Pathway-controlled division of C·A·B-droplets at RNA concentration condition (ii) in Figure 7a.

**Supplementary Movie 9:** Pathway-controlled division of C·A·B-droplets at RNA concentration condition (iii) in Figure 7a.

**Supplementary Movie 10:** Pathway-controlled division of C·A·B-droplets at RNA concentration condition (iv) in Figure 7a.
